# Effect of sampling volume on measurements of size and chemical homogeneity of MRI contrast agent FeraSpin™ R

**DOI:** 10.1039/d5na00463b

**Published:** 2026-02-10

**Authors:** Vittorio Maceratesi, Lavinia Rita Doveri, Nicholas Engel, Ester Cantoni, Piersandro Pallavicini, Chiara Milanese, Florian Sack, Nicole Gehrke, Andreas Briel, Nora Lambeng, Sarah Douri, Carine Chivas-Joly, Enrica Alasonati, Valentin de Carsalade du pont, Dimitrios Sapalidis, Marianna Gerina, Bruno F. B. Silva, Olivier Tache, William A. Lee, David J. H. Cant, Caterina Minelli, Christian Gollwitzer, Robin Schürmann, Yuri Antonio Diaz Fernandez

**Affiliations:** a Department of Chemistry, University of Pavia Via Taramelli 12 Pavia 27100 Italy ydf@unipv.it laviniarita.doveri@unipv.it; b Physikalisch-Technische Bundesanstalt Abbestr. 2–12 10587 Berlin Germany robin.schuermann@ptb.de; c Department of Biomedical and Inorganic Chemistry, Laboratoire National de Métrologie et D'Essais (LNE) 1 rue Gaston Boissier 75015 Paris France; d NanoPET Pharma GmbH D-10115 Berlin Germany; e Laboratoire National de Métrologie et D'Essais, LNE, DMSI – CARMEN Platform 29, Avenue Roger Hennequin 78197 Trappes France; f Center for X-ray Analytics, Empa, Swiss Federal Laboratories for Material Science and Technology Lerchenfeldstr. 5 9014 St. Gallen Switzerland; g Laboratory for Biomimetic Membranes and Textiles, Empa Lerchenfeldstr. 5 9014 St. Gallen Switzerland; h Laboratory for Biointerfaces, Empa Lerchenfeldstr. 5 9014 St. Gallen Switzerland; i Université Paris-Saclay, CEA, CNRS, NIMBE 91191 Gif Sur Yvette France; j Chemical and Biological Sciences Department, National Physical Laboratory Hampton road Teddington TW11 0LW UK

## Abstract

The use of iron oxide nanoparticles as contrast agents for magnetic resonance imaging (MRI) poses key questions regarding accurate determination of particle size and chemical composition within micro-heterogeneous systems. Here we present the first systematic study on homogeneity for particle size and chemical composition on the nanoparticle-based MRI contrast agent FeraSpin™ R, combining complementary analytical tools across a multidisciplinary consortium, clustered around the EURAMET project MetrINo. Our results indicate that, depending on the target measurand, sizing methods can provide consistent values for the particle colloidal diameter and for the size of the particle core, independently of the effective volume of the sample probed. Conversely, the evaluation of homogeneity for chemical composition depends on the length-scale of the sampling, in agreement with Benedetti–Pichler description of multicomponent systems. This case study highlights the importance of measurement length-scale for comparison and integration of data from complementary analytical methods, opening new avenues for standardization to support regulatory positioning of emerging nanomedicines.

## Abstract 2

The use of iron oxide nanoparticles as contrast agents for magnetic resonance imaging (MRI) poses key questions regarding accurate determination of particle size and chemical composition within micro-heterogeneous systems.

## Graphical abstract

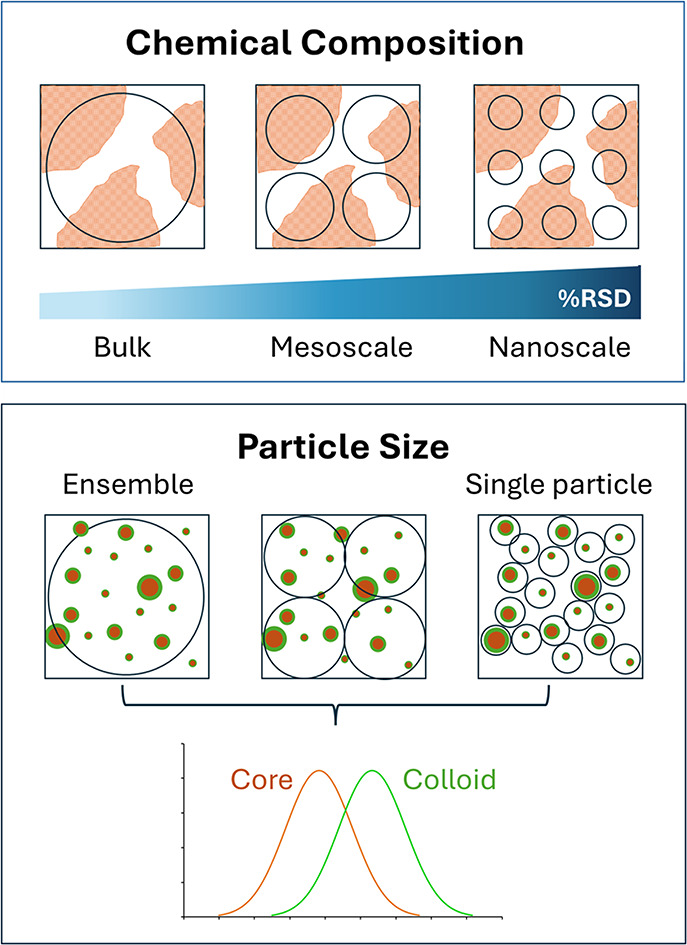

## Introduction

Nanotechnology has revolutionized the biomedical sector, improving diagnostic tools based on Magnetic Resonance Imaging (MRI) that exploit iron oxide nanoparticles (IONPs) as MRI contrast agents.^1,2^ A few IONPs formulations have been approved by the European Medicines Agency and United States Food and Drug Administration for clinical and pre-clinical trials on 
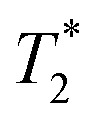
-weighted MRI, due to their ability to modify the transverse relaxation time of protons.^3–5^ The contrast efficiency of IONPs depends on the size of the magnetic core,^4^ while both MRI contrast and biodistribution are strongly influenced by the size of the particles.^1,6^ Consequently, the accurate measurement of particle core and particle shell size represents a priority target from technological and regulatory perspectives.

On the other hand, IONPs used for *in vitro* or *in vivo* studies must be biocompatible and stable in colloidal form. To ensure stability and biocompatibility, IONPs manufacturers often exploit biopolymer coatings to create core–shell structures, where the magnetically active core delivers the MRI contrast while the external coating prevents aggregation and ensures biocompatibility.^4,5^ The presence of the organic coating around the IONPs core introduces additional challenges for the precise characterization of these nanomaterials, increasing the bias between the size measured for the particle core and the effective size of the colloidal particles.^7^ Furthermore, within these multicomponent systems, accurate evaluation of chemical composition becomes critical to quantify contrast efficiency, control the dosage of the MRI contrast agent and lead to regulatory approval, supported by proper pharmacokinetics and pharmacodynamics data.^1,8^. These fundamental analytical questions stand at the forefront of scientific research and require the use of advanced, highly complementary methods able to provide insights on key measurands, on their uncertainty and on their homogeneity across IONPs samples.

Our work highlights the most recent efforts of MetrINo, a multidisciplinary EURAMET consortium,^9^ encompassing several metrology institutes, academic institutions and industry, working in synergy to address the grand challenge of standardization of analytical methods for the characterization of nanomedicines.^10^ The long-term ambition of MetrINo starts from the identification of candidate reference materials (RM) relevant to the context of nanomedicine and at an advanced stage of clinical or pre-clinical trials.^11^ Among the potential RM candidates, we identified FeraSpin™ R, a MRI contrast agent that is gaining increasing attention due to its high biocompatibility and excellent performance for preclinical diagnostic imaging.^12^ FeraSpin™ R is manufactured and commercialized by nanoPET Pharma GmbH as a complex formulation containing iron oxide nanoparticles stabilized by a carboxy-dextran-derived coating material.^13,14^ We present here the first systematic study on homogeneity for FeraSpin™ R particle size and chemical composition using several complementary methods. We show that sizing methods can consistently provide either the volume or intensity weighted hydrodynamic diameter of the colloidal particle or the number-weighted diameter of the particle core and these two measurands are systematically different, but individually consistent across relevant techniques. We also demonstrate that for micro-heterogeneous systems such as FeraSpin™ R, the concept of chemical homogeneity must be redefined, considering the effective sampling volume that each analytical method is able to measure, opening new avenues for data integration and comparison across different length scales.

## Results and discussion

### Estimating homogeneity of particle size by complementary analytical techniques

**Fig. 1:**
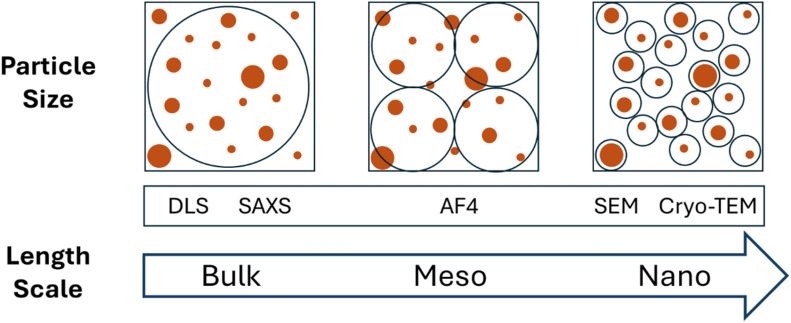
Analytical methods used to probe particle size on FeraSpin R formulations with the schematic representation of associated length scales.

Particle size and size distribution are critical quality attributes for FeraSpin™ R formulations, impacting bio-distribution and reliability of MRI data.^7^ The size of the metal-oxide core is key for delivering the magnetic properties, yet the hydrodynamic radius will determine the fate of the nanoparticles within living organisms. To assess the potential effects of sampling volume on the effectively measured particle size, we deployed a set of analytical techniques that covered a wide range of length scales, from bulk measurements probing large ensembles of particles to microscopy techniques able to probe the size of the single nanoparticles (Fig. 1).

**Fig. 2:**
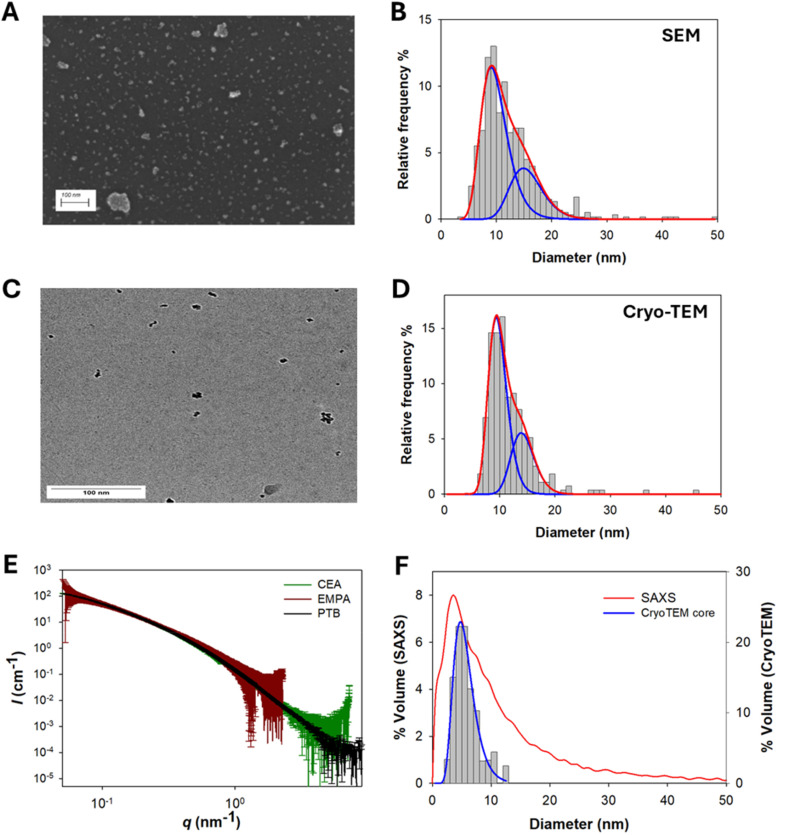
Particle size characterization of the original colloids of FeraSpin™ R formulations. (A) Representative SEM image showing particle morphology, scale bar 100 nm. (B) Size distribution (grey bars) obtained by SEM for particle diameter. (C) Representative cryo-TEM image, scale bar 100 nm. (D) Size distribution (grey bars) obtained by cryo-TEM for the geometrical diameter (average of two Feret diameters). (E) Comparison of SAXS data from CEA, EMPA, and PTB. The CEA and PTB data are nearly identical, while the small deviations to the EMPA data are within the 2*σ* uncertainties of the values. (F) Superposition of the size distributions of the sphere-equivalent diameter obtained by Monte-Carlo modelling of SAXS data (red line) and by cryo-TEM (grey bars) for particle core. Fitted data from PTB and CEA are presented in SI section C, Fig. SI 3b, c and d. Within frames B & D red lines represents the best fitting curve for two log-normal distributions, while blue lines depict the single log-normal components. Within frame F the blue line represents the best fitting curve for a log-normal distribution for cryo-TEM particle core data.

We initially investigated particle size and size distribution on FeraSpin™ R particles using Scanning Electron Microscopy (SEM), following an optimized sample preparation protocol that ensured good dispersion of the particles on poly-lysine coated silicon wafers. FeraSpin™ R samples showed a large number of small particles that appeared as quasi-spherical on the SEM images (Fig. 2B). We also observed larger particles that can be attributed to agglomerates or aggregates and that seem more heterogeneous in shape and size. We cannot exclude that some agglomeration occurred during sample preparation, and for this reason it is not possible to differentiate by SEM stochastic agglomerates formed during sample drying/spinning from aggregates that may be already present in the starting solution. Fig. 2B provides the number-based size distribution histogram built from the analysis of ∼600 particles. This histogram displays a monomodal distribution (red line) which can be modelled as the superposition of 2 log-normal distributions (blue lines). A continuous distribution with a range of sizes is observed, indicating a relatively small degree of polydispersity (FWHM 6.6 nm) for FeraSpin™ R. The ponderation between the two distributions led to 87% for the first contribution and 13% for the second one. The entire size distribution is dominated by the first peak, showing mean size around (12.1 ± 4.0) nm and a statistical mode at 9.6 nm.

FeraSpin™ R particle size was also investigated by cryogenic transmission electron microscopy (cryo-TEM, Fig. 2C). For this scope we adapted a cryogenic vitrification protocol, previously developed,^15^ based on grid-blotting and immediate plunge-freezing in liquid ethane (for details refer to Experimental Methods). This sample preparation method ensured good dispersion of FeraSpin™ R nanoparticles and accurate determination of particle size using the open-source software-package Fiji.^16^ The geometrical size for each object was calculated as the average of the two Feret diameters, including single particles and aggregates. The size distribution obtained by cryo-TEM (Fig. 2D) was monomodal and could be fitted by 2 log-normal size distributions, displaying average size at 11.8 nm and statistical mode at 9.7 nm, in agreement with the data discussed above for SEM. We expect cryo-TEM sample preparation to prevent agglomeration, therefore the aggregates observed in the micrographs (Fig. 2C) may be already present in the starting solution.

We also determined the hydrodynamic diameter of FeraSpin™ R original colloid using Dynamic Light Scattering (DLS). In this case the measurand was the intensity weighed harmonic average for particle diameter (*Z*-average size) that differs from the number-based size measured by electron microscopy. Using DLS we obtained moderately poly-dispersed, yet monomodal size distributions with mean diameters between 55 nm and 65 nm depending on the dispersion medium used, except for NaCl 0.9% that yielded an anomalously high particle diameter, probably due to colloidal stability issues discussed below. We also determined the *ζ*-potential in different dispersion media (Table SI1). The *ζ*-potential values obtained were all negative, suggesting that FeraSpin™ R particles display a negative surface charge independently of the dispersion media used, yet the absolute values decreased at higher ionic strengths. Reduction of particle *ζ*-potential in the presence of electrolytes is well described in the literature and could compromise colloidal stability.^1,17^ For this reason, we investigated the stability of FeraSpin™ R colloids over 24 h in four different matrices, confirming that the colloid was stable in pure water and in ammonium nitrate (see Table SI1), where it maintained a *ζ*-potential value lower than the stability threshold of −30 mV, PDI values below 0.3, and size distributions unchanged overtime. Conversely, FeraSpin™ R show some stability issues in pure NaCl 0.9%, showing an increase in size and polydispersity index over time, probably due to the reduced *ζ*-potential value in this high ionic strength matrix.

FeraSpin™ R size distribution was additionally investigated by Asymmetric Flow Field Flow Fractionation (AF4) coupled with UV, differential refractive index (DRI), and multi-angle light scattering MALS. Ammonium nitrate at 10^−4^ M, which has been shown to preserve the stability of FeraSpin™ R (see SI SI1), was used as the mobile phase. The main particle population eluted between 10 min and 30 min, as detected by MALS. In contrast, the UV and DRI detectors revealed a double-peak pattern, indicating the presence of an earlier eluting population of small size particles, eluting between 8 min and 12 min, that strongly absorbs at 300 nm but yields poor light scattering signal (SI Fig. SI2A). In this region, reliable MALS fitting was not possible due to poor signal quality or insufficient scattering intensity. It should be noted that the DRI signal showed a peak partially overlapping with the void peak, which is most likely an artefact. This signal corresponds to a transient pressure disturbance in the channel, occurring when the crossflow rate begins to decrease in power mode. This artefactual peak was also observed in the blank, confirming it is unrelated to the sample (Fig. SI2B). The size distribution calculated on the main population ranged from 32 nm to 144 nm, with a weight-average diameter of gyration of (56 ± 4) nm, leading to a diameter comparable to the values obtained by DLS (See SI Table SI1).

To assess size homogeneity of the particle core, we performed SAXS measurements at three independent institutes using different laboratory and synchrotron-based setups and on three different aliquots of one batch of FeraSpin™ R particles dispersed in water. The three datasets are in good agreement (Fig. 2E) and allowed us to verify a high degree of homogeneity across the different aliquots of the batch. The scattering curves of FeraSpin™ R are smooth and contain no distinctive scattering features, which indicates that the sample has a high degree of polydispersity. SAXS data were analysed using the Monte-Carlo regression package from the CEA software pySAXS that is based on McSAS^18^ and the fit results and representative results on the size distribution of FeraSpin™ R are presented in the histogram (Fig. 2B). Unlike other methods, such as Guinier or form-factor fitting, which use a size distribution function (*e.g.*, normal or lognormal), the Monte-Carlo approach can handle unknown and nonstandard size distributions and address the entire *q*-range. The McSAS analysis confirms a high degree of polydispersity of FeraSpin™ R and exhibits a pronounced skewness towards smaller sizes, characterised by a high number of particle diameters below 10 nm and a long, decaying tail extending up to approximately 60 nm and with very small contributions up to approximately 80 nm, whereas the significance of the latter is small due to the relatively high uncertainty of these small contributions. It is important to note that the uncertainties of the contributions for diameters > 30 nm are larger than the actual contributions, suggesting that in this size range SAXS size distribution is not reliable in this size range. The median diameter of the presented dataset is 8.1 nm and the mean diameter is 12.3 nm, in agreement with the SEM data presented above. The mode-maximum of the diameter-distribution is at 4.1 nm and the variance is 13.0 nm. When fitting a Gaussian to the distribution, we get a mean diameter *µ* = 5.4 nm and the standard deviation 2*σ* = 14.6 nm.

We would like to point out that the traceability of the Monte-Carlo method has not yet been demonstrated. However, the analysis of the scattering curves presented here, which are identical within their uncertainties, provide differences of the mean and median value of approximately 1 nm (±10%). These small discrepancies are likely primarily result from slightly different settings of the McSAS analysis rather than from actual differences in the experimental datasets. This aspect stresses how critical the definition of data processing parameters is and how variations of these parameters may emerge from a user-dependent choice of fitting parameter sets, selected as better options for heuristic reasons.

While interpreting the size distribution histogram obtained by SAXS, it should be noted that, in this particular case, we cannot distinguish between larger particles or aggregates of smaller particles using this technique. Additionally, due to the small electron density difference between the organic ligand shell and the water in which the particles are dispersed, the size obtained by SAXS mainly corresponds to the particle core sizes for individual particles or equivalent cluster diameter for particle aggregates. To evaluate the stability of FeraSpin™ R, the sample was measured in week 27 of 2023 and in week 5 of 2025 using the synchrotron-based setup. We observed no significant changes for the scattering curve over the 18-month period of this study (SI section C, Fig. SI3 a–d). The small deviation in overall intensity (<5%) is within the expected uncertainty for this measurement.

Additionally, using cryo-TEM imaging we were able to determine also the size of the particle core for every single particle, isolated or within larger aggregates, enabling the reconstruction of a size distribution for the core. Comparing this core size distribution obtained by cryo-TEM with SAXS data (Fig. 2F) we observed a good overlap of the main peak of the distributions, yet this high level of agreement may be coincidental, as the SAXS-derived distribution obtained by the Monte-Carlo approach is not unambiguous. The cryo-TEM distribution appeared narrower compared to SAXS distribution. This difference can be explained considering that cryo-TEM core size was calculated considering the single particles either isolated or inside the aggregates, while SAXS treat aggregates as single objects, with an equivalent cluster diameter, inducing a broadening of the SAXS size distribution.

In addition to the original FeraSpin™ R colloid, we also investigated size homogeneity on lyophilized samples, provided by nanoPET as solid aliquots obtained directly from FeraSpin™ R dispersions. This sample format could be particularly relevant in specific cases of clinical and analytical applications, considering the extended shelf-life and improved versatility for storage conditions.^19,20^ The lyophilized aliquots were redispersed in three different matrixes (water, saline solution and PBS), and the colloidal dispersions obtained were analysed by DLS. Interestingly, although the characteristic mean size was slightly shifted towards higher values, compared to the original FeraSpin™ R colloid, the size distributions remained monomodal with polydispersity indexes below 0.3 (See SI section D, Table SI2). Overall, our results suggest that FeraSpin™ R can be presented in a lyophilized format that is suitable for dispersion on demand for research purposes, opening new avenues to use this type of sample format for inter-laboratory comparison studies of key measurands, relevant to the nanomedicine community.

It is interesting to note that the characteristic size obtained from different analytical techniques clustered around two specific values, namely 12 nm and 57 nm (Table 1). These results can be rationalized considering the differences between the physical principles and the target measurands related to each analytical method. Cryo-TEM and SEM rely on measuring the size of single particles while the other methods measure an ensemble of particles for a given acquisition time. The sample volumes effectively measured varied among these different techniques, ranging from 1 mL for DLS, 25 µL for AF4-MALS, less than 1 µL for SAXS, and less than 7 µL for cryo-TEM (blotted and frozen) and SEM (deposited and spread by spin coating on PLL silicon wafer). However, the clustering observed around the two characteristic size values was not related to sample volume. To explain our results, we can consider that the techniques cryo-TEM and SEM provided number-weighted size distributions, which are more sensitive to smaller populations of particles. Conversely, the intensity-weighted analysis of DLS and AF4-MALS, tends to skew the size distribution towards higher values due to their weighting of larger particles by scattering intensity. These differences may be further enhanced by the limit of detection for each technique. While SEM can measure particle sizes down to a few nm, MALS shows poor sensibility below 10 nm, leading to a systematic bias on the determination of particle size. Additionally, DLS accounts for the effective hydrodynamic size of the particles, that will consider the extended radius of the organic coating on colloidal FeraSpin™ R particles, that may collapse under the dry conditions of SEM measurements and will also not contribute much to the size values determined by SAXS, due to poor X-ray scattering properties of the organic material. We can therefore conclude that even though the target measurands are different for different techniques, the two families of methods (SEM, cryo-TEM, and SAXS on one end, and DLS and AF4-MALS at the other end) provide complementary information regarding the mean size of the particle core and the intensity-weighted mean size for the colloidal dispersions of FeraSpin™ R particles. Similarly, polydispersity data obtained from complementary techniques may lead to inconsistent results, and intercomparison of different methods must be considered with caution.

**Table 1:** Characteristic particle diameter obtained by complementary techniques

Technique	SEM	Cryo-TEM	SAXS	DLS	AF4-MALS
Measurand	Geometrical diameter^a^	Geometrical diameter^b^	Particle core diameter^c^	Hydrodynamic diameter^d^	Gyration diameter^e^
Mean size (nm)	12.1 ± 4.0	11.8 ± 4.8	12.3 ± ∼1.0	57.1 ± 1.0	56.8 ± 8.0

a Number-based average diameter measured by SEM with expanded uncertainty (*k* = 2), derived from parameter propagation.

b Number-based average diameter measured by cryo-TEM as the mean of two Feret diameters for each object.

c Mean diameter measured by SAXS with estimated uncertainty (±10%).

d Intensity-weighted *Z*-average diameter obtained by DLS using 10^−4^ M NH_4_NO_3_ as dispersant, with repeatability.

e Intensity-weighted gyration diameter measured by AF4-MALS with mobile phase 10^−4^ M NH_4_NO_3_, with uncertainty.

Using SAXS, the extracted diameters of the different aliquots are identical within the uncertainties (approx. ±10%) of the results from the Monte-Carlo analysis. Similarly, using DLS repeatability data, we could estimate that the variability for the intensity-weighted mean colloidal diameter was below 0.5%. These estimations of homogeneity in particle size include uncertainty contributions from the different analysis methods and instrumental settings; therefore, the actual size homogeneity of FeraSpin™ R may be overall better than 3%, which is remarkable for an intrinsically poly-dispersed formulation of industrial and clinical relevance.

### Estimating chemical homogeneity by complementary techniques

**Fig. 3:**
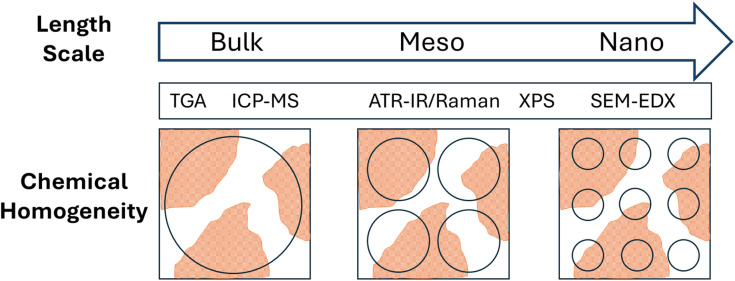
Analytical techniques used to assess chemical homogeneity of FeraSpin R formulations with the schematic representation of associated length scales.

Chemical homogeneity is another critical quality attribute of nanomedicine and theragnostic formulations.^10,21^ In the specific case of FeraSpin™ R, used as preclinical MRI contrast agent suitable for intravenous injection,^22^ accurate evaluation of chemical composition represents a key step towards quantitative delivery of effective doses, reproducible MRI data acquisition and stronger regulatory positioning. FeraSpin™ R consists of nanoparticles of iron oxide (predominantly *y*-Fe_2_O_3_) stabilized by a carboxy-dextran-derived coating.^13^ The active MRI component is the iron oxide core, while the coating ensures stability and biocompatibility.^4,5^ In the case of freeze-dried FeraSpin™ R samples, we observed a tendency to absorb moisture from the environment, perceived even at necked eye by the transition from a fine powder to a sticky gel when the samples were exposed to moisture for hours. This hygroscopic effect led to a three-component system, containing iron oxide, organic coating and loosely-bond water molecules. Within the following sections we explore different analytical methods for evaluating chemical homogeneity of FeraSpin™ R samples, discussing advantages and limitations of each method. With these techniques we probed different compositional measurands and covered a wide range of length scales: from macroscopic samples to micrometric and nanometric spot-size acquisitions typical of micro-spectroscopy methods (Fig. 3).

Thermo-gravimetric analysis (TGA) under reactive atmosphere is a traceable analytical tool, providing direct quantification of the weight content for volatile, non-volatile organic and inorganic components within complex formulations. The ability to analyze relatively large aliquots of the target materials (in the range of milligrams), provides a direct route to examine chemical homogeneity in aliquot dimensions relevant to *in vitro* and *in vivo* experimentation.

**Fig. 4:**
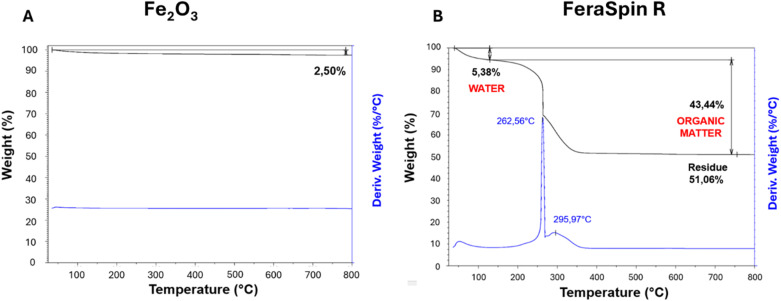
Representative thermo-gravimetric analysis (TGA) data for: (A) control iron oxide nanopowder; (B) FeraSpin™ R powders. Additional TGA data is presented in Fig. SI5a and b. TGA experiments were performed in 5 different aliquots of the samples, and chemical homogeneity was estimated calculating the relative standard deviations of moisture, non-volatile organic and inorganic components.

We performed TGA analysis in dry air under controlled flow conditions. To exclude contributions from iron oxidation, we performed control experiments with magnetite, an iron oxide with mixed Fe(ii) and Fe(iii) states, obtaining negligible weight changes close to experimental error (see SI section E, Fig. SI5a and b). Similarly, we run TGA analysis on control Fe(iii) oxide samples, obtaining losses in weight below 3% mainly due to the release of moisture (Fig. 4A). Conversely, when FeraSpin™ R samples were analyzed using TGA (Fig. 4B), we observed a different profile, starting by an initial decrease in weight of about 5% below 150 °C that can be attributed to evaporation of loosely-bond water molecules. Subsequently the samples underwent a net loss of weight over 40% between 200 °C and 400 °C, consistent with the complete combustion of non-volatile organic molecules in air. The residual weight remained stable up to 800 °C, allowing for the determination of the inorganic fraction at around 50% in weight. TGA experiments on different aliquots of FeraSpin™ R also allowed the estimation of sample homogeneity (expressed as the relative standard deviations of the weight fractions across five distinct aliquots), obtaining variabilities for the non-volatile organic, inorganic residue and moisture component within ± 0.37%, ± 0.31%, and ± 0.64%, respectively. Interestingly, the variability of the ratio between inorganic and organic components (excluding moisture) was below 0.3%, confirming that FeraSpin™ R samples are chemically homogeneous.

Chemical homogeneity was also investigated using inductively coupled plasma mass spectrometry (ICPMS) on liquid samples of FeraSpin™ R, after microwave-assisted acid mineralization (further details in Experimental Section and Table SI3). ICPMS is the gold standard analytical technique for the determination of metals in solution and therefore offers a direct route for estimation of chemical homogeneity of FeraSpin™ R in terms of variability of iron content within different aliquots. With this method we obtained chemical homogeneity within 0.7%, confirming that iron content on FeraSpin™ R formulation is highly homogeneous across different aliquots.

We also investigated chemical homogeneity using two vibrational spectroscopy methods, namely infrared and Raman spectroscopy. Infrared spectroscopy represents a fast, versatile and fit-for-purpose technique for routine quality control of chemical homogeneity within production pipelines, particularly in the version attenuated total reflectance infrared (ATR-IR) spectroscopy, able to analyze solid samples deposited directly on the ATR crystal, without specific sample preparation. However, extracting quantitative information on chemical composition from ATR-IR data may be challenging for solid powders, due to the fact that the absolute intensity of the IR peaks depends on the coverage of the ATR crystal and on the granulometry of the sample: irrespective of the mass of sample used, only the fraction of sample within a couple of microns from the ATR crystal can be analyzed.^23^ Despite this limitation, chemical homogeneity can be assessed by ATR-IR comparing the relative intensities of the IR peaks of the different components within the sample.

**Fig. 5:**
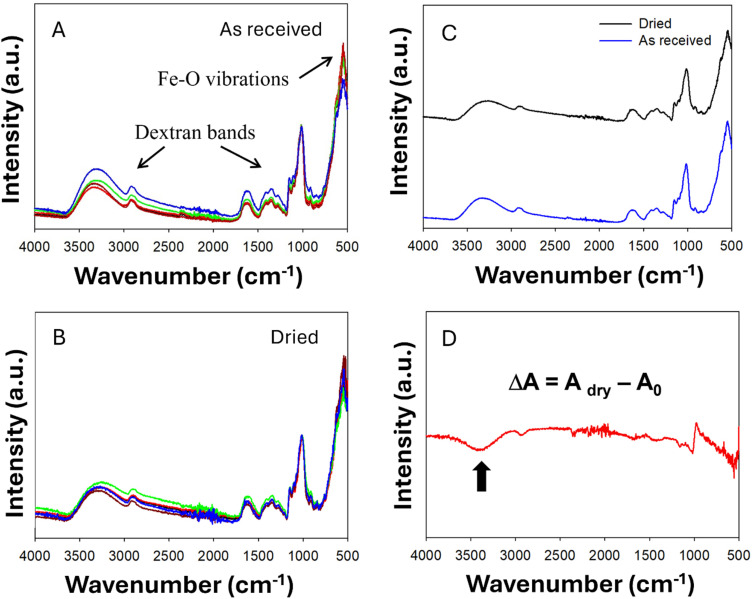
Representative attenuated total reflectance infrared (ATR-IR) data on f FeraSpin™ R: (A) different aliquots as received; (B) different aliquots after drying at 105 °C overnight; (C) average spectra before and after drying; (D) difference spectrum highlighting the loss of water molecules with a black arrow.

ATR-IR spectra of different aliquots of FeraSpin™ R, presented in Fig. 5, display a broad band below 554 cm^−1^, that can be attributed to Fe–O vibrational modes present in other iron oxides (See SI Fig. SI6).^24,25^ Additional peaks between 700 cm^−1^ and 2000 cm^−1^can be assigned to the organic coating, while the broad band with a maximum around 3400 cm^−1^ is related to the stretching mode of hydroxyl groups, including those of the capping agent and of residual water molecules present in the solids. The distinct positions of these peaks allowed for the normalization of the single spectra, setting to unity the peak at 1012 cm^−1^ related to the capping agent to unity. Spectra normalized by the peak attributed to the organic capping agent are effectively plots of intensity ratios between the different components and the variability of these normalized peaks across different sample aliquots provide direct evidence of sample homogeneity, considering that all other factors are compensated by normalization of the peaks.

After normalization of the ATR-IR spectra, we observed some variability of the relative intensities of IR peaks for iron oxide and for –OH groups. The variability in the OH region was considerably reduced when the samples were thermally pre-treated overnight at 105 °C to remove the moisture contribution, yet a significant OH band remained visible on the dried samples, due to the presence of OH groups in the non-volatile capping agent. Evidence of successful removal of loosely-bound water molecules was also observed by computing the difference-spectrum (Fig. 5D) obtained by subtracting the average normalized spectra before and after drying. We observed a consistent reduction of peak intensity around 3400 cm^−1^ after drying. Conversely, the peak of iron oxide below 554 cm^−1^ remained unchanged, and the main peak of the organic capping agent displayed only a slight shift upon drying. These results confirm that an additional drying step on the freeze-dried samples may improve chemical homogeneity, particularly in terms of moisture content. Additionally, by calculating the variance of ATR-IR data at wavenumbers representative of Fe–O vibrational modes, we inferred that the fluctuation of chemical composition of freeze-dried FeraSpin™ R samples remained within 3% in terms of inorganic to organic ratios.

**Fig. 6:**
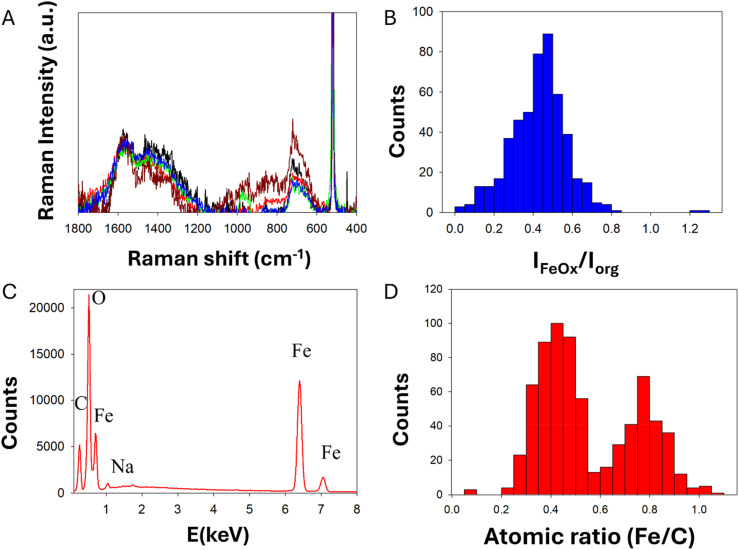
Micro-spectroscopy data for dried FeraSpin™ R samples: (A) representative normalized µ-Raman spectra; (B) µ-Raman histogram for the intensity ratio of the peaks characteristic of iron oxide and of the organic coating; (C) representative SEM-EDX spectra; (D) SEM EDX histogram of the atomic ratio between Fe and C.

A similar approach was used to investigate chemical homogeneity by µ-Raman micro-spectroscopy. In this case the lyophilized FeraSpin™ R powder, as received, was not suitable for direct microscopy analysis, due to the presence of subtle flakes of the solid material that were not mechanically stable under laser illumination. For this reason, the samples were redispersed in distilled water and different aliquots were re-deposited on a Si wafer and allowed to dry overnight at room temperature. This process produced solid films with a thickness of approximately 20 µm, estimated by optical microscopy. On these films we were able to record over 500 Raman spectra at random locations across different aliquots. For our instrument configuration, the lateral spot size of the laser was 2.1 µm with a confocal aperture slit of 50 µm that allowed for the entire vertical section the sample film to be analyzed (*i.e.* sampling volume below 100 µm^3^). All the spectra showed distinct peaks that were attributed to the organic coating and to Fe–O vibrations (Fig. 6 A, additional spectral data for peak attribution is presented in SI Fig. SI7).

Although the absolute intensity of the peaks varied across different locations of the sample, the ratio of the peaks attributed to Fe–O and to the organic coating provided a measure of chemical homogeneity, indicating that the variability of the inorganic to organic ratio was below 6%, in agreement with ATR-IR data. Interestingly, the ratio of the Raman peaks for iron oxide and for the organic component across the entire date set followed a monomodal statistical distribution with characteristic mode at 0.48 and a certain level of tailing and asymmetry (excess kurtosis 6.32 and skewness 0.42, Table SI4), supporting the fact that FeraSpin™ R samples are highly homogeneous even at the micrometric scale probed by the Raman microscope.

Chemical homogeneity was also probed by X-ray photoelectron spectroscopy (XPS) on solid films obtained from FeraSpin™ R dispersions with a sample preparation method similar to the one described above for µ-Raman. XPS is a surface sensitive technique able to provide quantitative elemental composition, however it is essential to minimize the deposition of adventitious material during sample preparation, since surface contamination at trace levels may significantly bias XPS measurements. We adopted two sample preparation strategies to validate our results: one set of samples were dried in air, covering the surface with a cap to avoid dust deposition. Another set of samples was dried under vacuum, to prevent deposition of adventitious material. These two sets of samples were analyzed by XPS during a unique working session to reduce any other sources of measurement variability, and led to identical elemental compositions, within experimental uncertainty (See SI section J, Tables SI7 and SI8). This result suggests that air-drying and vacuum-drying sample preparation methods are equivalent for the analysis of FeraSpin™ R samples by XPS. Interestingly, in addition to iron, carbon and oxygen, we observed the presence of sodium, which could act as counterion for the capping agent. We also observed a residual amount of silicon due to the sample support used during the experiments. On the other hand, the atomic ratio between iron and carbon (Fe/C) obtained by XPS was considerably lower than the range expected from TGA data (*i.e.* 50% in weight of FeraSpin™ R is iron oxide). This apparent discrepancy can be explained considering the different chemical environments probed by each analytical technique. While TGA is a bulk method probing the entire sample, XPS probe a few first nanometers of the sample surface. Under our XPS measurement conditions, the lateral spot size was 300 µm × 700 µm with an estimated depth below 5 nm. The core–shell structure of FeraSpin™ R nanoparticles leads to a preferential sampling of the organic coating at the surface, generating a bias in the absolute determination of Fe/C ratios by XPS.

Despite this analytical bias, XPS data allowed us to estimate chemical homogeneity by calculating the relative standard deviation of the atomic ratio between iron and carbon (Fe/C) on three different aliquots. We expect iron-oxide and the dextran-based stabilizer to be the only sources of iron and carbon, respectively. Therefore, the %RSD for the Fe/C ratio across different sample aliquots will directly reflect chemical homogeneity in this two-component system, estimated to be below 7% by XPS analysis (full data set and representative XPS spectra are presented Fig. SI8a, b, c, d, e and f).

FeraSpin™ R dried aliquots were also analyzed by SEM-EDX to obtain the full elemental composition on random locations of the samples (Fig. 6C and D). This data also revealed the presence of the expected elements carbon, oxygen, iron and a residual amount of sodium, probably acting as counterion for the particle negative surface charge. We calculated the ratio between iron and carbon (Fe/C) for each EDX point as a measure of chemical composition, obtaining a bimodal distribution with the main mode at 0.42 and a distinct secondary mode at 0.77, suggesting that at the length scale of SEM analysis the sample showed local fluctuations of composition that clustered in two main micro-phases. It is important to note that the sensitivity of EDX to light chemical elements like C is limited and may lead to a systematic bias on the determination of Fe/C ratios. Furthermore, SEM-EDX has the smallest sampling volume among all the techniques investigated here, determined by a penetration depth of the electron beam at 20 kV lower than 2 µm (*i.e.* sampling volume ∼4 µm^3^). Despite these facts, the main statistical mode for the atomic ratio Fe/C on SEM-EDX data was close to the mode of the ratio *I*_FeO*x*_/*I*_org_ obtained by µ-Raman, which is a remarkable similarity, and may indicate that both, elemental composition and vibrational spectroscopy, are good predictors of chemical composition for FeraSpin™ R. Using SEM EDX data, we estimate an overall variability of chemical composition around 12%, the highest of all the techniques investigated here.

Comparing chemical homogeneity data obtained from these four complementary methods, we can conclude that, irrespective of the specific measurand use (*i.e.* ratio of two vibrational peaks, Fe/C elemental ratio, ratio of inorganic-to-organic weight fraction), FeraSpin™ R samples were highly homogeneous in chemical composition. We also observe a general trend, correlating the level of homogeneity observed by each method with the effective size of the sample analyzed. While TGA deals with milligram aliquots and provided the best chemical homogeneity data below 1%, ATR-IR and Raman were able to measure mesoscopic portions of the samples with intermediate homogeneity results around 6%, and SEM-EDX, relying on the smallest bulk sampling volumes among all the techniques investigated here, provided the highest level of chemical variability at around 12% (see Table 2). This trend is not surprising and can be partially explained with the Benedetti–Pichler equation for a two-component system.^26^ In this approximation, a micro-heterogeneous sample can be represented by an ensemble of two populations of “particles” with different densities (*d*_1_ & *d*_2_), different chemical compositions (*C*_1_ & *C*_2_) and total particle number concentration *N*_P_ (*i.e.* number of particles per gram of sample). If we assume that one of the particle types has a normalized abundance equal to *f* within the sample, the relative standard deviation for the chemical composition determined experimentally can be written as:1
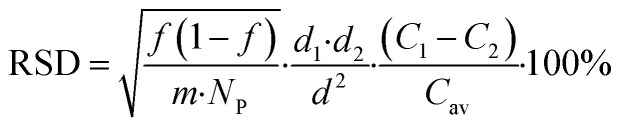
where *d* and *C*_av_ are the average density and average composition of the entire sample, and *m* is mass of the aliquot analyzed. From eqn (1) we can predict that the compositional RSD measured on a small aliquot of a micro heterogeneous sample will scale with the inverse square root of the effective mass of that aliquot. The smaller the mass analyzed (*i.e.* smaller sampling volume), the higher the variability observed, which is precisely what we obtained while comparing compositional data from bulk methods (*i.e.* TGA) with surface sensitive techniques with mesoscopic probing volumes (*i.e.* ATR-IR, µ-Raman) and with nanoscale spectroscopic data (*i.e.* SEM-EDX). Using eqn (1), we can estimate the order of magnitude for the quantity of mass (*m*_*i*_) effectively analyzed by the analytical method indexed “*i*” with compositional RSD_*i*_ (%). Knowing that for TGA the order of magnitude of sample mass and the RSD measured were ∼1 mg and 0.3%, respectively, we obtain:2
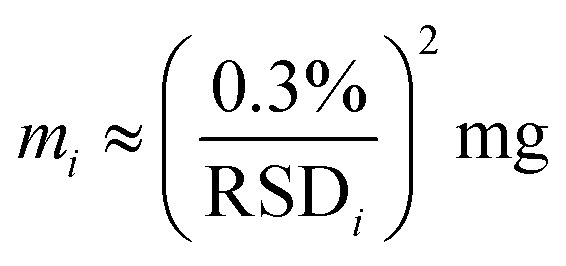
With this approximation, we estimated that the sample aliquots effectively measured by ICPMS as 180 µg, the sample size analyzed by ATR-IR and µ-Raman were ∼3 µg, by XPS was ∼2 µg, while the mass probed by SEM-EDX was around 0.6 µg. These values are in good agreement with the sampling volumes discussed above.

**Table 2:** Sample size *vs.* chemical homogeneity

Length scale	Bulk		Meso		Nano
Technique	TGA	ICPMS	IR/Raman	XPS	SEM-EDX
Sample size	∼1 mg	∼180 µg	∼3 µg	∼2 µg	∼0.6 µg
Chemical homogeneity (RSD)	0.30%	0.70%	6%	7%	12%

Our results suggest that the relevance of chemical homogeneity obtained at different length scales in the context of nanomedicine research must be commensurate to the scope of the measurement and to the level of correlation with clinical and biological data. Bulk measurements such as TGA are fit for purposes to fulfill regulatory requirements in terms of chemical homogeneity of injectable aliquots. On the other hand, interpreting microscopy data related to clinical assays may require a better understanding of compositional fluctuations within smaller sampling volumes. Similarly, extrapolating chemical composition from micro-spectroscopy methods to bulk samples may jeopardize the assessment of sample homogeneity within micro- and nano-heterogeneous formulations. Our work confirms the need for a multidisciplinary approach, encompassing a wide range of analytical techniques and covering different length-scales to tackle the grand challenge of chemical homogeneity assessment for emerging nanomedicines.

In addition to the length-scale effect discussed above, quantitative deconvolution of different uncertainty contributions to the measured chemical homogeneity would require the quantification of all other sources of uncertainty for each specific analytical technique, but this ambitious task is beyond the scope of the work presented here. Nevertheless, our results demonstrate that in the case of chemical composition, the choice of the analytical method is critical to assess the relevant level of homogeneity for a complex micro- or nano-heterogeneous formulation, highlighting the importance of effective sampling volumes and length-scales on the interpretation and comparison of data from complementary analytical techniques.

## Conclusions

In this work we investigated the colloidal size, particle core size and chemical homogeneity of the MRI contrast agent FeraSpin™ R. The sample is composed of iron oxide nanoparticles with an overall particle diameter around 12 nm as determined by SAXS, cryo-TEM and SEM. Analysis of different aliquots provided consistent results with a variability below 3%, suggesting that FeraSpin™ R formulations are highly homogeneous in size. Hydrodynamic sizes were also evaluated by MADLS and MD-AF4, obtaining values that ranged from 50 nm to 94 nm, depending on the dispersion matrix used. *ζ-*potential measurements confirm that storage in aqueous media with low ionic strength ensures the best colloidal stability in terms of electrostatic stabilization. Variability of hydrodynamic size across different aliquots of FeraSpin™ R remained below 0.5%. We also assessed the chemical composition by thermogravimetry and by different spectroscopic techniques, allowing the evaluation of chemical homogeneity in terms of inorganic and organic components. In this case we observed a trend between the estimated chemical homogeneity and the effective sampling volume for each technique. We obtained homogeneity better than 99% for bulk measurements (*e.g.* TGA, ICPMS), above 90% for mesoscopic techniques (*e.g.* ATR-IR, µ-Raman, XPS), and around 85% for nanoscale spectroscopic methods (*e.g.* SEM-EDX). Our results highlight the relevance of effective sampling volumes and length-scales for interpretation and comparison of data obtained from complementary techniques on complex nanomedicine formulations.

## Experimental

### Materials

FeraSpin™ R samples, in the form of lyophilized powder and as colloidal dispersion (20 mM of iron) were provided by nanoPET Pharma GmbH (Berlin, Germany). Iron(iii) oxide nanopowder (Fe_2_O_3_, 50 nm based on BET) and Iron(ii, iii) oxide nanopowder (Fe_3_O_4_, 97%, 50 nm to 100 nm based on BET) were purchased from Merck Life Science s.r.l. (Milan, Italy) and were used as received without any further purification. Phosphate buffered saline (PBS) tablets, sodium chloride (NaCl, ACS reagent ≥99,0%), ammonium nitrate (NH_4_NO_3_) were also obtained from Merck Life Science s.r.l. (Milan, Italy) were dissolved in MilliQ water (Millipore system by Merck). Prior to measurement each buffer was filtered through 0.2 µm cellulose acetate membrane filters (Carlo Erba Reagents s.r.l (Milan, Italy). Nitric acid (≥99 999%) was obtained from Merck Life Science s.r.l. (Milan, Italy) and hydrochloric acid (>99,0% purity) was purchased from Carlo Erba Reagents s.r.l (Milan, Italy).

Dry powders of FeraSpin™ R nanoparticles were obtained by freeze drying of water-based particle dispersions. Apart from the carboxydextran-based coating polymer, no other capping agents were present within the dispersion. To remove this excess coating polymer from the dispersion, centrifugal ultrafiltration was performed using Sartorius® Centrisart 1 centrifugal ultrafiltration devices (Sartorius Lab Instruments GmbH, Göttingen, Germany) with a molecular weight cutoff (MWCO) of 300 kDa. 1 mL of FeraSpin™ R particle dispersions was centrifuged for 20 min at 1800 g. Excess supernatant was discarded and replenished with equal amounts of MilliQ water. After an equilibration time of 2 min this washing procedure was repeated four times. Following purification, nanoparticle dispersion depleted of free polymer was immediately frozen in liquid nitrogen for 2 min to 5 min. Samples were either directly subjected to lyophilization or stored at −80 °C for later use. The drying process was carried out using a laboratory freeze dryer Alpha 1-2 LSCbasic (Martin Christ Gefriertrocknungsanlagen GmbH, Osterode am Harz, Germany) in accordance with the manufacture's operation protocol. After completion, dried samples were weighed and sealed with Parafilm® (Heathrow Scientific, Chicago, USA) for subsequent transport.

### FeraSpin™ R characterization

#### Multi-angle dynamic light scattering

Batch MADLS measurements were performed at 25 °C on a Zetasizer Ultra instrument (Malvern Panalytical) equipped with a 633 nm laser, operating at 10 mW power and in three angles detection mode: back scattered detection (173° scattering angle), side (90°) and forward (13°). The *ζ-*potential was measured using folded capillary zeta cell (dts1070, Malvern) using the Smoluchowski model for high or moderate ionic strengths and the Huckle model for pure water. The size, the polydispersity index (PDI) and *ζ-*potential results were obtained by averaging 3 consecutive measurements. We reported the results for the size at the back scattered angle (*Z*-average 173°), the polydispersity index (PDI) and the *ζ*-potential.

FeraSpin™ R colloidal dispersions were analyzed after 1 : 15 dilution (1.33 mM of iron) from the stock solution provided by NanoPET Pharma GmbH in four different matrices (water, 0.9% (w/w) NaCl solution, phosphate saline buffer 0.01 M-PBS, and ammonium nitrate 10^−4^ M-NH_4_NO_3_) and stored at 4 °C. The size was measured at 1 hour, 6 hours, and 24 hours after sample preparation, while the zeta potential was measured at 1 hour and 24 hours.

FeraSpin™ R lyophilized samples were prepared at 1.3 mM (based on total Fe concentration) by dissolving the solids in the appropriated media (water, 0.9% (w/w) NaCl solution, and PBS). To facilitate dispersion, a three-minute sonication was performed using Bandelin Sonorex Ultrasonic baths. For each matrix, five aliquots were prepared, and the size was obtained by averaging nine consecutive measurements at an angle of 173° and the *ζ-*potential by averaging three consecutive measurements. Size and *ζ-*potential were measured immediately after sample preparation.

#### Fourier-transform infrared spectroscopy (FTIR-ATR) spectroscopy

FeraSpin™ R, Fe_2_O_3_ and Fe_3_O_4_ nanopowders were analyzed with a Nicolet IS20 spectrometer (Thermo Fisher Scientific, Waltham, MA, 104 USA), equipped with the ATR accessory consisting of a diamond crystal (FTIR-ATR) for the analysis of solid samples in reflectance mode. IR spectra were acquired in reflectance mode over a range from 4000 cm^−1^ to 500 cm^−1^, with a spectral resolution of 4 cm^−1^ and collecting a total of 32 scans. A background spectrum was collected before each sample acquisition to exclude environmental signals.

Five aliquots of FeraSpin™ R powder were sampled and analyzed as received, while five additional aliquots were dried for 24 hours in an oven at 105 °C and then cooled in a desiccator before IR analysis. All raw IR data were converted to absorbance and normalized for the absorbance value at 1012 cm^−1^. Fe_2_O_3_ and Fe_3_O_4_ samples were analyzed under the same conditions described for FeraSpin™ R. No drying process was performed in this case. All raw IR data were expressed in absorbance and were not normalized at the source.

#### Thermogravimetric analysis (TGA)

Thermogravimetric Analysis (TGA) was performed using a TA Q5000 instrument (USA) on FeraSpin™ R, Fe_2_O_3_ and Fe_3_O_4_ nanopowders. A sample amount between 2 mg and −5 mg was placed in a platinum crucible and heated in air flow. The heating rate was set to 10 °C min^−1^ from room temperature to 800 °C. Air was used during the analysis at a flow rate of 10 cm^3^ min^−1^ to maintain an oxidizing environment throughout the test and ensure the complete combustion of the organic coating at 800 °C. Tests were performed in quadruplicate and processing was performed using the TA Universal Analysis 2000 software version 4.5A.

#### Inductively coupled plasma mass spectrometry (ICP-MS)

The total concentration on Fe was analysed by external calibration with a single quadrupole ICP-MS (iCapQ from Thermo Fischer Scientific, Courtaboeuf, France) equipped with a sea-spray borosilicate nebulizer. A quartz cyclonic spray chamber was used as sample introduction system. Prior to analysis, 50 µL of sample was mineralised with 2 mL of nitric acid (69% HNO_3_ (w/w)). The mixture was heated in a microwave reactor (CEM Discover SP). The temperature was increased from 20 °C to 190 °C in 10 min and kept at 190 °C during 15 min. The sample was then diluted in 2% HNO_3_. The ICP-MS conditions for the characterisation of Fe are given in Table 3.

**Table 3:** Instrumental parameters for the characterisation of total Fe by ICP-MS

Parameter	
Dwell time	100 ms
Acquisition time	80 s
Nebulizer gas flow	≈1.0 L min^−1^
Sample input flow	0.5 mL min^−1^
Gas mode	He
Collision/reaction gas flow	5 mL min^−1^
*m*/*z*	^56^Fe, ^57^Fe

#### Raman spectroscopy

Sample preparation involved dissolving 1 mg of the powder in 40 µL of water. Subsequently, 5 µL of the sample solution was deposited onto silicon substrates, allowed to dry, and the process was repeated four times to ensure sufficient sample coverage. Raman spectra of FeraSpin™ R, Fe_2_O_3_ and Fe_3_O_4_ samples were acquired using a DXR2xi Raman imaging microscope (ThermoFisher Scientific, Waltham, MA, USA) with excitation at 532 nm and 1 mW laser power. A 10X objective was used for imaging. Each individual spectrum was obtained by averaging 12 accumulations with 5 s acquisition time each. For Fe_2_O_3_ and Fe_3_O_4_ samples, 10 representative spectra were collected in random locations. To assess homogeneity of FeraSpin™ R, spectra were obtained in mapping mode, with 100 spectra taken from five distinct zones (for a total of 500 spectra). Mapping was conducted over an area with a fixed step size of 140 µm × 100 µm. FeraSpin™ R spectra were normalized to the 1570 cm^−1^ peak and the baseline was corrected. Using the TQ Analyst software (ThermoFisher Scientific, Waltham, MA, USA), the inorganic-to-organic ratio was calculated by taking the ratio of the peak height at 700 cm^−1^ (associated with Fe oxides^27^) to the peak height at 1570 cm^−1^ (related to the organic component).

#### Scanning electron microscopy (SEM)

Drops of lyophilized FeraSpin™ R Samples prepared for Raman spectroscopy (3.4) were analyzed at University of Pavia with Scanning Electron Microscope (SEM) Zeiss EVO MA10 (Carl Zeiss, Oberkochen Germany) coupled with an Xmax microprobe (Oxford) (SEM-EDX). EDX analysis was performed at high voltage (20 kV), in high vacuum, at room temperature, at a magnification of 1000 and recording 10 spectra in 10 regions for 6 aliquots.

Colloidal FeraSpin™ R samples were imaged at LNE with a Zeiss ULTRA-Plus equipped of a Field Emission Gun (FEG) microscope and in-Lens SE detector. All images have been recorded through secondary electrons collected by an InLens detector. The sample was tested at an acceleration voltage of 3 kV at working distance of 3 mm and with magnifications fixed at 100 000. The Platypus® software developed by Pollen Metrology^28^ was used to recognize particles, leading to a size distribution. The histogram of the SEM data was fitted with an analytical size distribution using the software R-Studio with a program developed by the LNE statistics team, which uses the well-known statistical method of Maximum-Likelihood estimation.^29^ The size distributions measured by EM-based analysis were fitted by a mix-log normal function. The output mean diameter and standard deviation were determined, together with the 95%-confidence interval for both parameters. The uncertainty on the particle size determined by SEM was estimated as described in SI section K. In the present study, particle size distributions were based on a total number of particles counted fixed at 600. The FeraSpin™ R particles provided from Nano-PET were diluted 10 times in milliQ water and deposited on a silicon wafer coated with PLL (Poly-l-Lysine) to functionalize the substrate surface and to promote an efficient attachment between particle and substrate. For getting a good dispersion, a drop of suspension was drop-casted and then spin-coated to prevent agglomeration and promote dispersion on the substrate.

#### Small-angle X-ray scattering (SAXS)

SAXS measurements were performed at three institutes with different setups.

Synchrotron-based SAXS measurements were carried out at the four-crystal monochromator beamline of the Physikalisch-Technische Bundesanstalt at the BESSY II synchrotron radiation facility in Berlin-Adlershof using the SAXS facility of the Helmholtz-Zentrum Berlin. The samples were irradiated with a beam of synchrotron radiation with an energy of 8 keV and a cross-sectional area of 150 × 400 µm^2^ at the position of the capillaries. The radiation scattered by the samples was recorded by a vacuum-compatible Pilatus 1M detector,^30^ which in week 27, 2023 was located (2864 ± 1) mm and (5063 ± 1) mm behind the samples in the short and long configurations, respectively. In week 5, 2025 it was located, almost identically, (2864 ± 1) mm and (5064 ± 1) mm behind the samples in the short and long configurations, respectively.

Laboratory-based SAXS measurements were performed on a Bruker Nanostar, Bruker AXS GmbH, (Karlsruhe, Germany), using a Kα-line of a micro-focused X-ray Cu source (Incoatec) with an X-ray energy of 8.0 keV. The samples were measured in a sealed quartz capillary under vacuum. The beam was further passed through 2D beam shaping MONTEL optics and a collimation block, with a 0.3 mm scatterless pinhole serving as the beam-defining aperture, resulting in a beam diameter of approximately 0.4 mm at the sample position. An evacuated flight tube (∼0.01 mbar) between the sample and the detector reduces absorption and air scattering. The scattered intensity was recorded with a 2D MikroGap technology-based detector (VÅNTEC-2000, Bruker AXS) with 2048 × 2048 pixels, each 68 × 68 µm^2^ in size. The sample-detector distance was set to 147 cm (as determined from measuring silver behenate powder as a standard) achieving a resolvable *q*-range of 0.05 nm^−1^ ≤ *q* ≤ 1.8 nm^−1^. The exposure time for the measurement was 3 h. The transmitted fraction of the beam was obtained from a home-made semi-transparent beam stop.

In addition, SAXS measurements were performed on a Xenocs (Grenoble, France) XEUSS 3.0 instrument, using an X-ray Cu (8 keV) and Mo (17 keV) source, and a Dectris Eiger3 1M detector. The instrument is beamstopless, meaning that the direct beam is integrated during the acquisition, in order to obtain absolute intensities directly. The thickness of the capillaries (same batch, same manufacturer) was estimated at 0.12 cm by measuring the scattered intensity of a water capillary. Different configurations were used with different sample to detector distances (1.8 m, 0.9 m, 0.37 m). Images were acquired at 600 s on each configuration. The data were set in the absolute scales and corrected by the dark noise of the detector, subtracted by the water capillary, and merged from different configurations. We used the CEA pySAXS software for the data treatment.

For all SAXS measurements, the 2D-scattering images were reduced to 1D-data by azimuthal integration using proprietary software (PTB), the Bruker software DIFFRAC.EVA (Bruker AXS, version 4.1) (EMPA), and using pySAXS (CEA).

Each colloidal nanoparticle (NP) solution was filled into separate glass capillaries, borosilicate for CEA (from the same manufacturer and same batch) and PTB (WJM Mark-tubes, 1 mm diameter, 0.01 mm thickness) and quartz (Hilgenberg Mark-tubes, 0.01 mm thickness of about 200 µL volume) for EMPA and sealed before measurement. The filled capillaries were then loaded into the vacuum chambers and irradiated.

All SAXS data was corrected for transmission, background corrected (subtraction of scattering intensity from the capillary and solvent) and normalized by the capillary thicknesses. In the McSAS analysis, the SAXS data was fitted using 300 spheres with diameters in the range 0.1 nm < *d* < 80 nm. 100 repetitions were calculated whereas one repetition consists of a reduced chi square optimization and the different results of the individual repetitions are used to estimate the uncertainties of each of the 300 sphere diameters. Multiple sets of those fits were performed using varying parameters, *e.g.*, with varying *q*-ranges to ensure the robustness of the fit results. While for the fit result presented in the main article the range 0.06 nm^−1^ < *q* < 3 nm^−1^ was used, the full *q*-range was 0.05 nm^−1^ < *q* < 10 nm^−1^, see SI for the fit result. The results are in agreement.

#### Multi-detector asymmetrical flow field-flow fractionation (MD-AF4)

The MD-AF4 applied in this study was an AF4 system (AF2000 Postnova Analytics, Landsberg, Germany) coupled to multiangle laser light scattering (MALS) (DAWN HELEOS II, Wyatt Technology, Santa Barbara, USA) equipped with 18 angles, UV (SPD-20A, Shimadzu, Kyoto, Japan) and differential refractive index (DRI) (RID 20A, Shimadzu) detectors. An analytic AF4 channel (335 mm × 60 mm) metal-free (Postnova Analytics) was used. The carrier was prepared by dissolving NH_4_NO_3_ (Sigma-Aldrich, USA) in ASTM Type I ultrapure water (from a Milli-Q system Q-POD Element (Millipore, USA) and filtering through a 0.1 µm filter (RC, Postnova Analytics).

The channel out-let flow rate was 0.5 mL min^−1^. The focusing and elution conditions were as follows: (i) focusing conditions: inlet flow rate of 0.2 mL min^−1^, focusing flow rate of 2.3 mL min^−1^ and crossflow rate of 2 mL min^−1^, focusing time of 5 min; (ii) elution conditions: 2 min of constant crossflow rate at 2 mL min^−1^, followed by a power (0.1) gradient crossflow rate decreasing from 2 mL min^−1^ to zero in 40 min and a final zero-plateau of 20 min. Main optimised parameters are reported in Table 4. MALS data treatment was performed using the Berry model of first degree and the Astra Software (Wyatt Technology). For MALS data treatment and for recovery calculation the UV detector was used.

**Table 4:** Optimised parameters for AF4-UV-DRI-MALS

Membrane	PES 10 kDa (postnova analytics)
Spacer	350 µm
*λ*_max_ UV (nm)	300
Eluent phase	NH_4_NO_3_ 10^−4^ M
Sample treatement	Dilution 1 : 10 in mQ water
Injected volume	25 µL
Separation method	Power (0.1) gradient

#### CryoTEM experiments

Cryo-TEM was performed using a Talos Arctica (Thermo Fisher Scientific) operated at 200 kV and equipped with a Ceta 16M detector (Thermo Fisher Scientific). Briefly, 3 µL of FerapinR nanoparticles were placed onto previously glow-discharged Quantifoil R1.2/1.3 Cu 300-mesh grid at 30 mA for 30″in a GloQube (Quorum Technologies). Sample vitrification was carried out using a Mark IV Vitrobot (Thermo Fisher Scientific) at 4 °C and 100% humidity, with blotting times of either 2 or 10 seconds prior to plunge-freezing in liquid ethane. Images were acquired at a nominal magnifications of 190 000×, corresponding to a pixel size of 0.0535 nn/pixel with a defocus of −3.0 and −4.0 µm, and at magnification 120 000× corresponding to a pixel size of chem 0.086 nm/pixel with a defocus of −2.0 and −4.0 µm. Image processing and data analysis were performed using Fiji.^16^

#### X-ray photoelectron spectroscopy (XPS)

For the sample preparation, 1 mg of the powder was weighed and dissolved in 40 µL of water to obtain a homogeneous solution. From this mixture, 5 µL were deposited onto silicon substrates (1 × 1 cm). A total of six samples were prepared: three were left to dry under ambient air, while the other three were dried under vacuum in a desiccator to minimize exposure to atmospheric moisture. In parallel, an additional 5 µL of pure water were deposited on separate substrates prepared under the same conditions. XPS spectra were recorded on three different aliquots for each sample preparation method with an AXIS Supra^+^ instrument from Kratos Analytical Ltd, using a Monochromated Al Kα (1486.6 eV) and a spot size of 300 µm × 700 µm. Additional experimental details are provided in SI Table SI8.

## Author contributions

Y. A. D. F. was the leading author, contributing data curation and interpretation, supervision of UniPV team, preparation of the first draft and following revisions, and coordination of the entire work. V. M. performed ATR-IR, TGA, and MADLS analysis and contributed to data curation and interpretation. L. R. D. performed µ-Raman and SEM-EDX MADLS experiments, contributing to data curation and interpretation, as well as supervision and investigation. E. C. performed AF4-MALS analysis. P.P contributed funding to support the research. C. M. contributed with TGA analysis and funding. N. L. contributed with SEM experiments, sampling methodology, investigation, and data curation. S. D., performed SEM experiments and contributed to writing, reviewing & editing, and data curation. C. C.-J., scientific contribution to writing, reviewing & editing, as well as validation and supervision of LNE CARMEN team. V. C. contributed with experiments and data curation for AF4. E. A. supervised LNE team for AF4 and contributed with data curation and interpretation. N. E. performed SAXS data acquisition and analysis from CEA and PTB, preparation of SAXS figures, first draft of PTB SAXS section, edited SAXS contributions from PTB, CEA, EMPA, performed manuscript proof reading. C. G. contributed to SAXS data analysis and writing of SAXS sections. R. S. performed SAXS data acquisition, and contributed to writing SAXS section, as well as coordinating MetrINo project. O. T. performed SAXS analysis on CEA SAXS data and wrote CEA SAXS subsection. D. S. contributed with SAXS data acquisition, processing, and curation; authored EMPA SAXS section. M. G. contribute SAXS data curation; and text to EMPA SAXS section. B. S. provided SAXS data curation; contributed to EMPA SAXS section; and supervision of EMPA team. W. A. L., D. J. H. C., and C. M. contributed with XPS analysis and XPS data interpretation. All authors reviewed and approved the manuscript.

## Conflicts of interest

FeraSpin™ R is a trademark of nanoPET Pharma GmbH. All samples used in this work were provided by nanoPET Pharma GmbH within the context of the Euramet project MetrINo. The analyses were performed by independent research teams that are not affiliated with nanoPET Pharma GmbH. All other authors have no conflict of interest to declare.

## Abbreviations

AF4: Asymmetrical flow field-flow fractionation
DLS: Dynamic light scattering
DRI: Differential refractive index
EDX: Energy-dispersive X-ray spectroscopy
FTIR-ATR: Fourier-transform infrared spectroscopy
FWHM: Full width half maximum
IONPs: Iron oxide nanoparticles
MADLS: Multiangle dynamic light scattering
MALS: Multiangle laser light scattering
MRI: Magnetic resonance imaging
MWCO: Molecular weight cutoff
PBS: Phosphate saline buffer
PLL: Poly-l-Lysine
RM: Reference materials
SAXS: Small-angle X-ray scattering
SEM: Scanning electron microscopy
TGA: Thermogravimetric analysis

## Supplementary Material

NA-008-D5NA00463B-s001

## Data Availability

Data supporting this article have been included as part of the supplementary information (SI). Supplementary information is available. See DOI: https://doi.org/10.1039/d5na00463b.
