# Supplementary material for: Effect of sampling volume on measurements of size and chemical homogeneity of MRI contrast agent FeraSpin™ R
Source: Nanoscale Adv. 2026 Feb 10;8(10):3136–50. doi: 10.1039/d5na00463b (PMC12915306; doi:10.1039/d5na00463b)
Supplement: NA-008-D5NA00463B-s001 [file NA-008-D5NA00463B-s001.pdf]

Supplementary Information (SI) for Nanoscale Advances.
This journal is © The Royal Society of Chemistry 2026

Supporting Information

Title: Effect of sampling volume on measurements of size and chemical homogeneity of MRI contrast agent FeraSpin™ R

Authors: Vittorio Maceratesi, Lavinia Rita Doveri,* Nicholas Engel, Ester Cantoni, Piersandro Pallavicini, Chiara Milanese, Florian Sack, Nicole Gehrke, Andreas Briel, Nora Lambeng, Sarah Douri, Carine Chivas-Joly, Enrica Alasonati, Valentin de Carsalade du pont, Dimitrios Sapalidis, Marianna Gerina, Bruno F.B. Silva, Olivier Tache, William A. Lee, David J.H. Cant, Caterina Minelli, Christian Gollwitzer, Robin Schürmann,* Yuri Antonio Diaz Fernandez*

Corresponding authors:

Yuri Antonio Diaz Fernandez (ydf@unipv.it),

Robin Schürmann (robin.schuermann@ptb.de),

Lavinia Rita Doveri (laviniarita.doveri@unipv.it)

A)- DLS data on the original FeraSpin™ R colloid

Table SI1- DLS data on the original FeraSpin™ R colloid as function of time

**H_2O**	**1 h**	**6 h**	**24 h**
**Size MADLS (nm)**	69 ± 2	69 ± 3	66 ± 4
**Size DLS 173° (nm)**	61.3 ± 0.3	62 ± 2	61 ± 2
**PDI**	0.202 ± 0.004	0.191 ± 0.002	0.196 ± 0.005
**ζ Potential (mV)**	-46 ± 2	/	-41.5 ± 0.4
**NaCl 0,9%**	**1 h**	**6 h**	**24 h**
**Size MADLS (nm)**	102 ± 12	205 ± 14	154 ± 123
**Size DLS 173° (nm)**	94 ± 2	150 ± 15	380 ± 40
**PDI**	0.274 ± 0.004	0.66 ± 0.07	0.49 ± 0.05
**ζ Potential (mV)**	-10.7 ± 0.6	/	-13 ± 3
**PBS 0.01 M**	**1 h**	**6 h**	**24 h**
**Size MADLS (nm)**	75 ± 2	92 ± 8	98 ± 10
**Size DLS 173° (nm)**	65 ± 1	85 ± 4	91 ± 3
**PDI**	0.23 ± 0.02	0.369 ± 0.007	0.41 ± 0.04
**ζ Potential (mV)**	-16 ± 3	/	-18 ± 2
**$NH_4NO_3 10^{-4} M$**	**1 h**	**6 h**	**24 h**
**Size MADLS (nm)**	**-**	**-**	**-**
**Size DLS 173° (nm)**	57.1 ± 0.1	58.2 ± 0.4	57 ± 1
**PDI**	0.20 ± 0.01	0.21 ± 0.03	0.212 ± 0.001
**ζ Potential (mV)**	-37 ± 3	/	-19 ± 2

Note: Each value is the average of three measurements with the respective standard deviations.

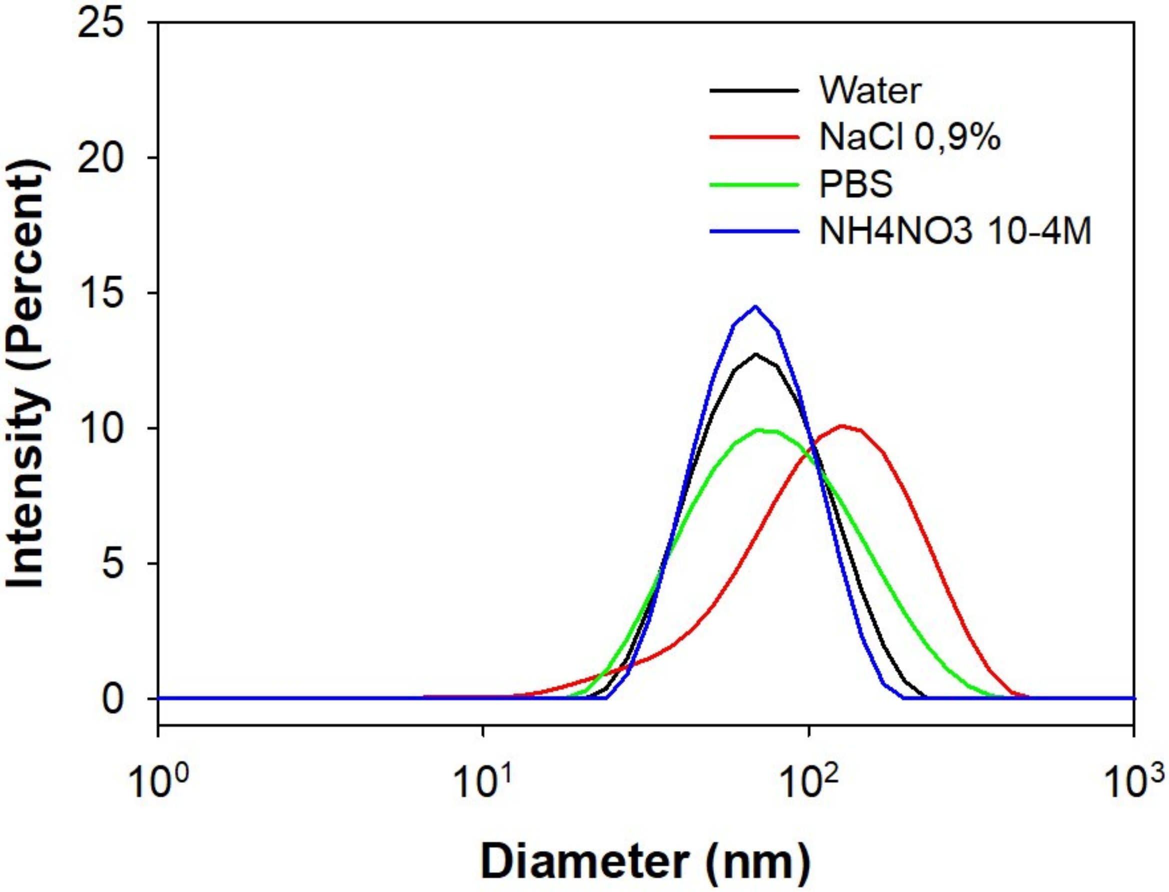

Figure SI1- Size distribution obtained by DLS in different dilution media (water, sodium chloride 0.9 %, and phosphate saline buffer (PBS) 0.01 M and NH4NO3 10-4 M)

B)- Multi-detector AF4 analysis of FeraSpin ™ R particles

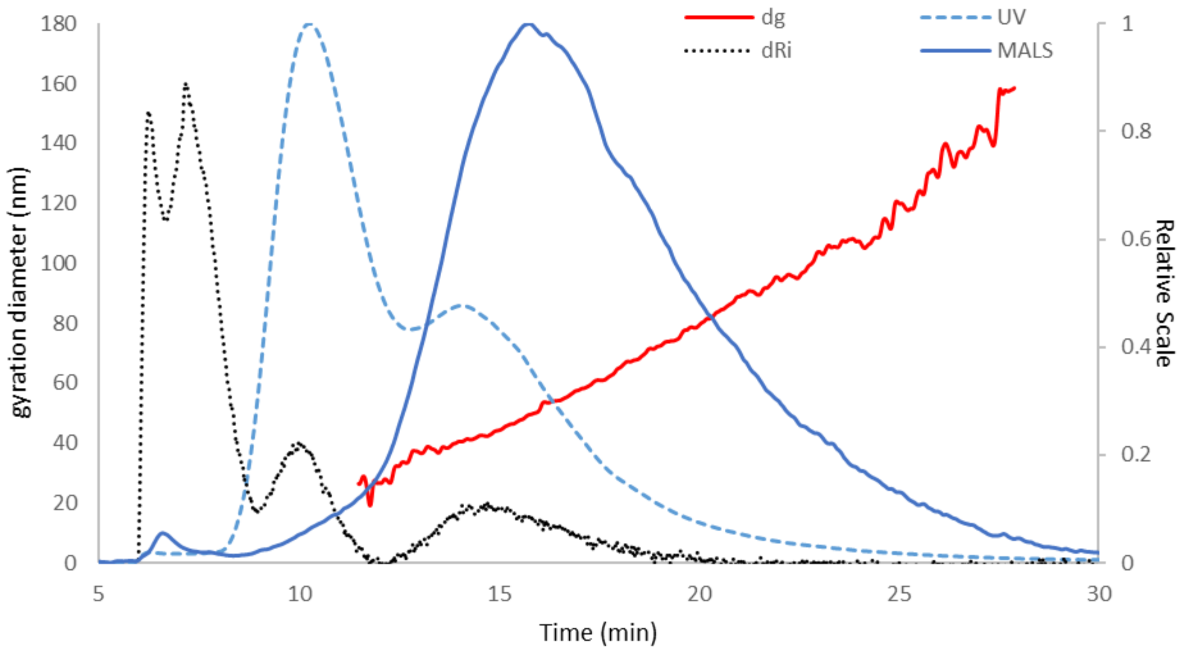

Figure SI2a- Analysis of FeraSpin ™ R by AF4-UV-DRI- MALS. Left axis: gyration diameter distribution. Right axis: relative scale for UV, DRI and MALS signals.

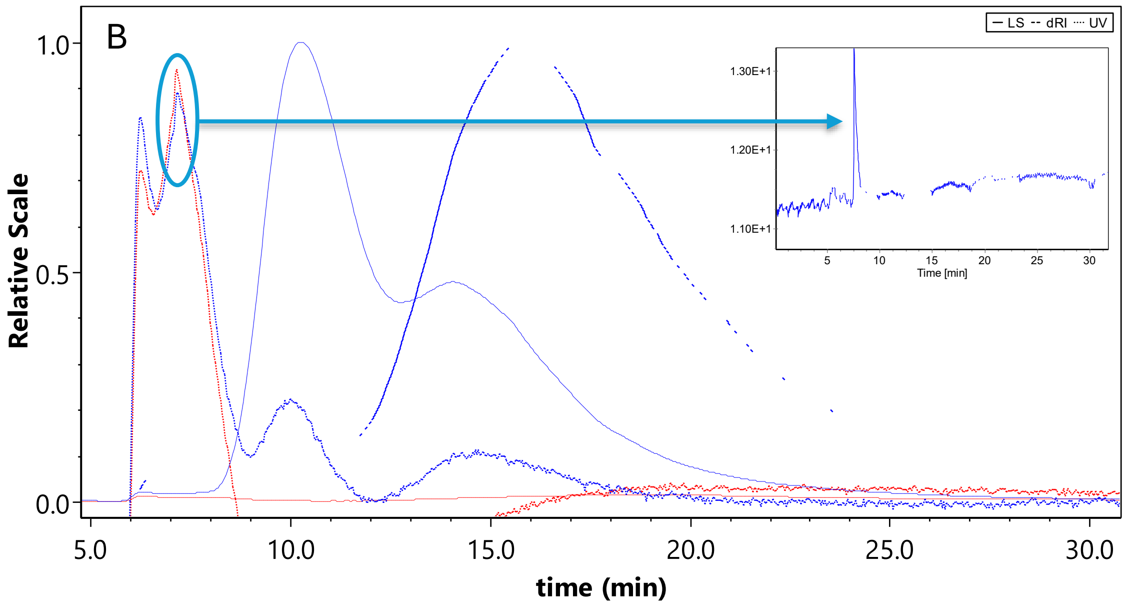

Figure SI2b- FeraSpin ™ R (in blue) *vs* blank analysis (in red) (relative scale): DRI signal shows a peak partially overlapping with the void peak, which corresponds to a transient pressure disturbance in the channel, occurring when the crossflow rate begins to decrease in power mode. The inlet shows the pressure measured inside the AF4 channel during the elution.

C) Additional SAXS data on FeraSpin ™ R

To present the CEA, EMPA, and PTB SAXS data together, we scaled them and subsequently subtracted a constant to streamline the amount of background.

Using the synchrotron-based setup FeraSpin ™ R was measured once in week 27, 2023 and once in week 5, 2025 to evaluate the stability of the sample and no significant changes of the scattering curve could be observed over the approximately 18-month period of this study. The small deviation in overall intensity (<5 %) is within the expected uncertainty for this measurement.

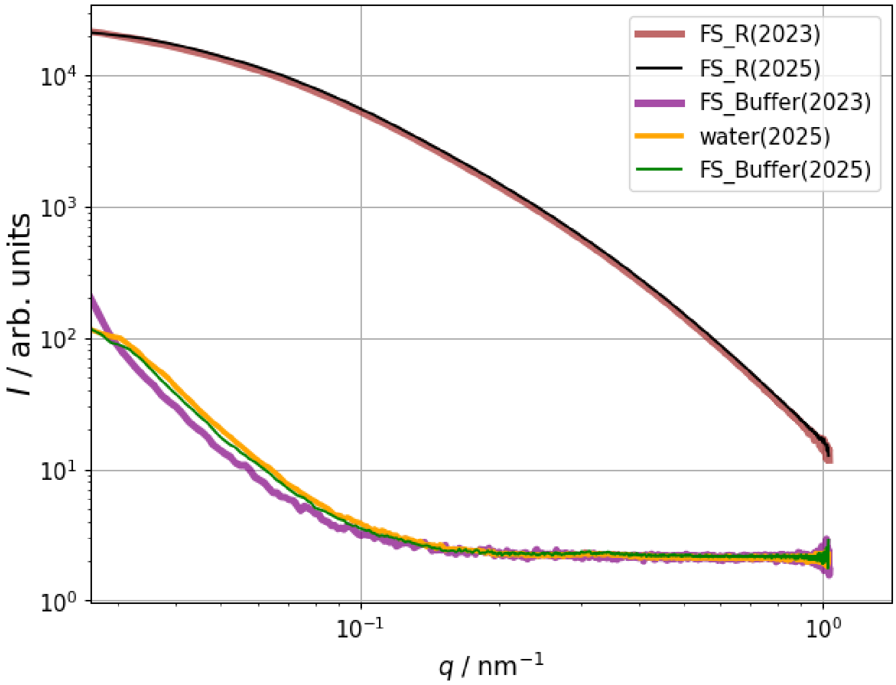

Figure SI3a- PTB SAXS data of FeraSpin ™ R (brown and black), the buffer (purple and green) were measured at PTB in week 27, 2023 and remeasured in week 5, 2025. The scattering curve of the buffer is dominated by the water scattering, compare orange scattering curve of water measured in week 5, 2025.

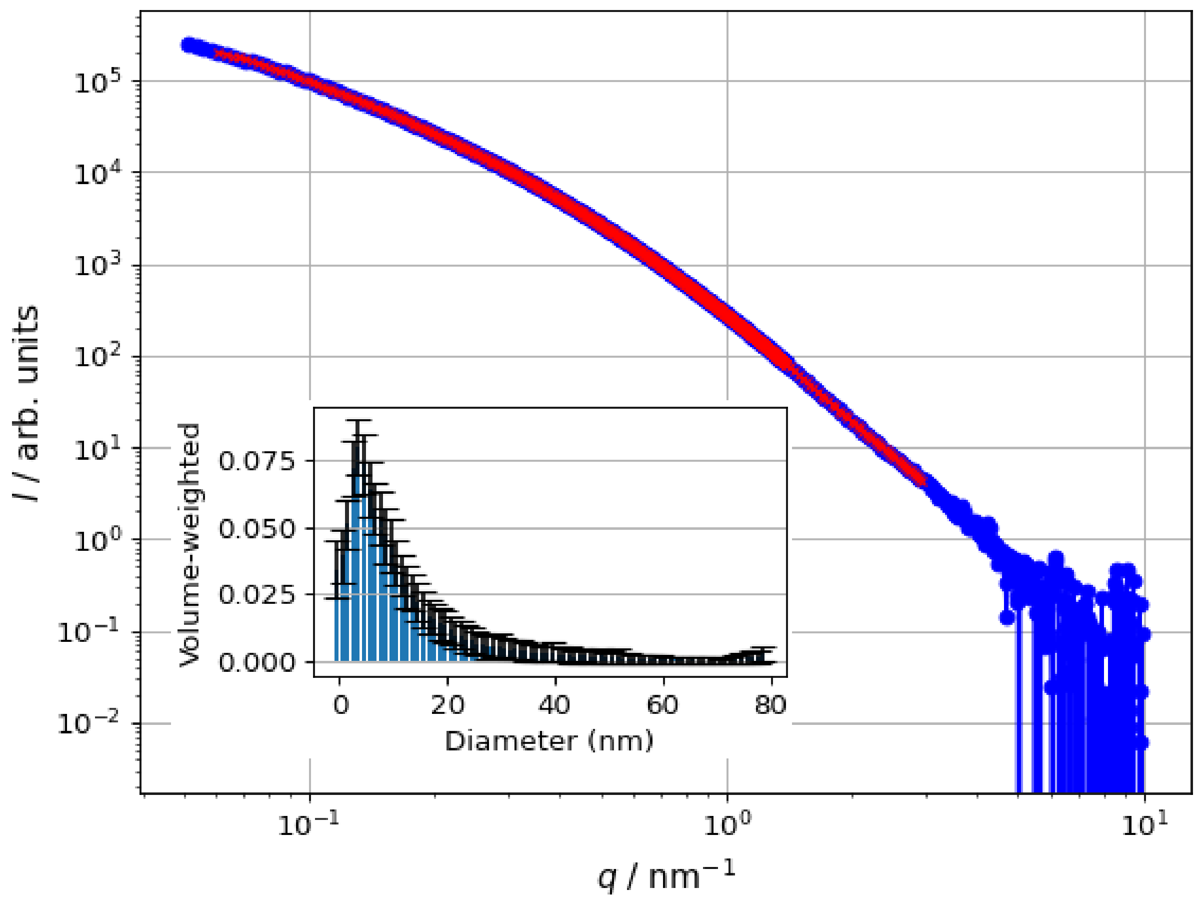

Figure SI3b- The PTB SAXS data is presented together with its fit, fitted using pysaxs, and in the inset the particle size distribution histogram (volume-weighted vs diameter). The median diameter is 8.1 nm, the mean diameter is 12.3 nm, the maximum of the distribution is 4.1 nm, and the variance is 13.0 nm. When fitting a Gaussian to the distribution we get a mean value $\mu = 5.4$ nm with a standard deviation $2\sigma = 14.6$ nm, as presented in the main article.

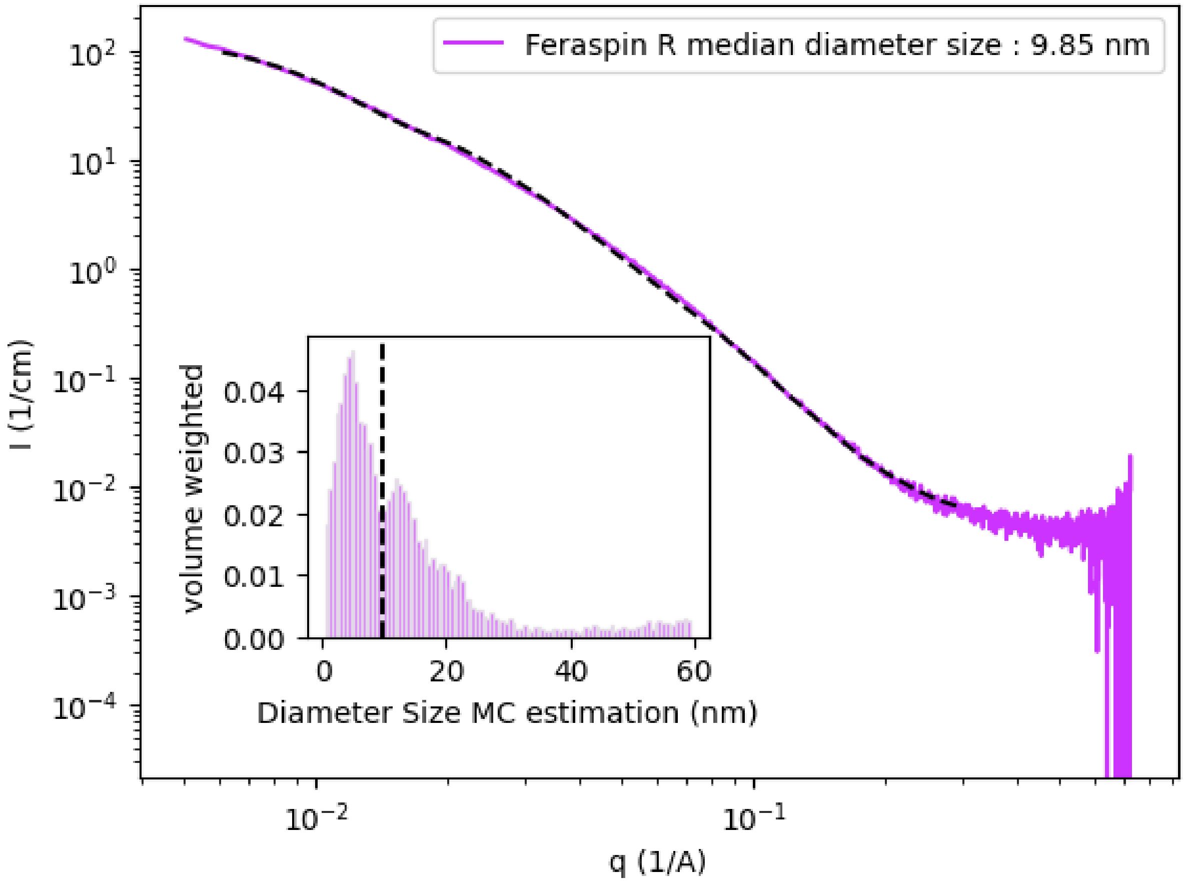

Figure SI3c- CEA SAXS data of FeraSpin ™ R, fitted using pysaxs. The median diameter is 9.9 nm and the mean diameter is 13.2 nm.

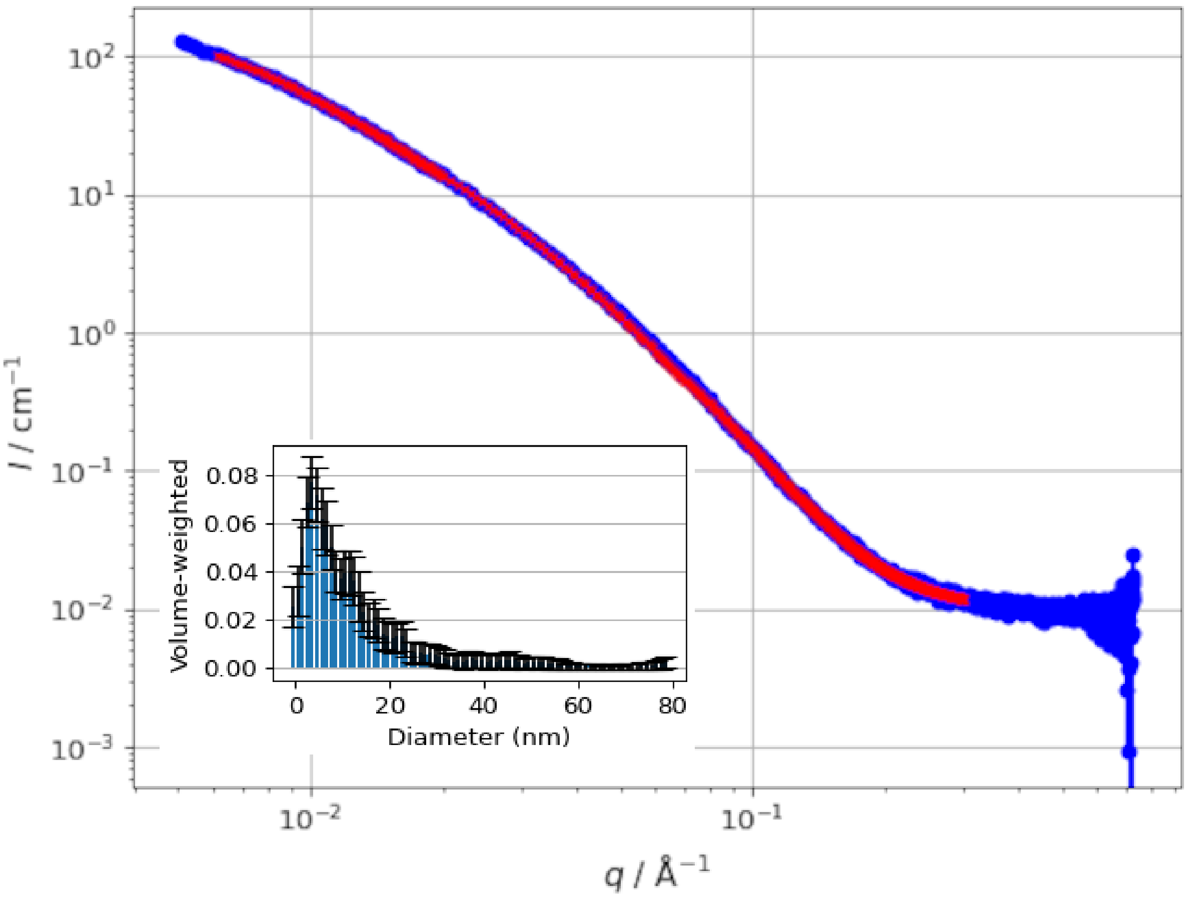

Figure SI3d- For Comparison: CEA SAXS data fitted by PTB using pysaxs and slightly different settings than CEA used → 9.1 nm median diameter.

The median diameter is 9.1 nm, the maximum of the distribution is at 4.1 nm, and the variance is 13.0 nm, identical to the results from the main article. The mean diameter is 12.8 nm (12.3 nm in the main article). When fitting a Gaussian to the distribution, we get a mean value μ = 5.9 nm (5.4 nm in the main article) and the standard deviation 2σ = 15.8 nm (14.6 nm in the main article).

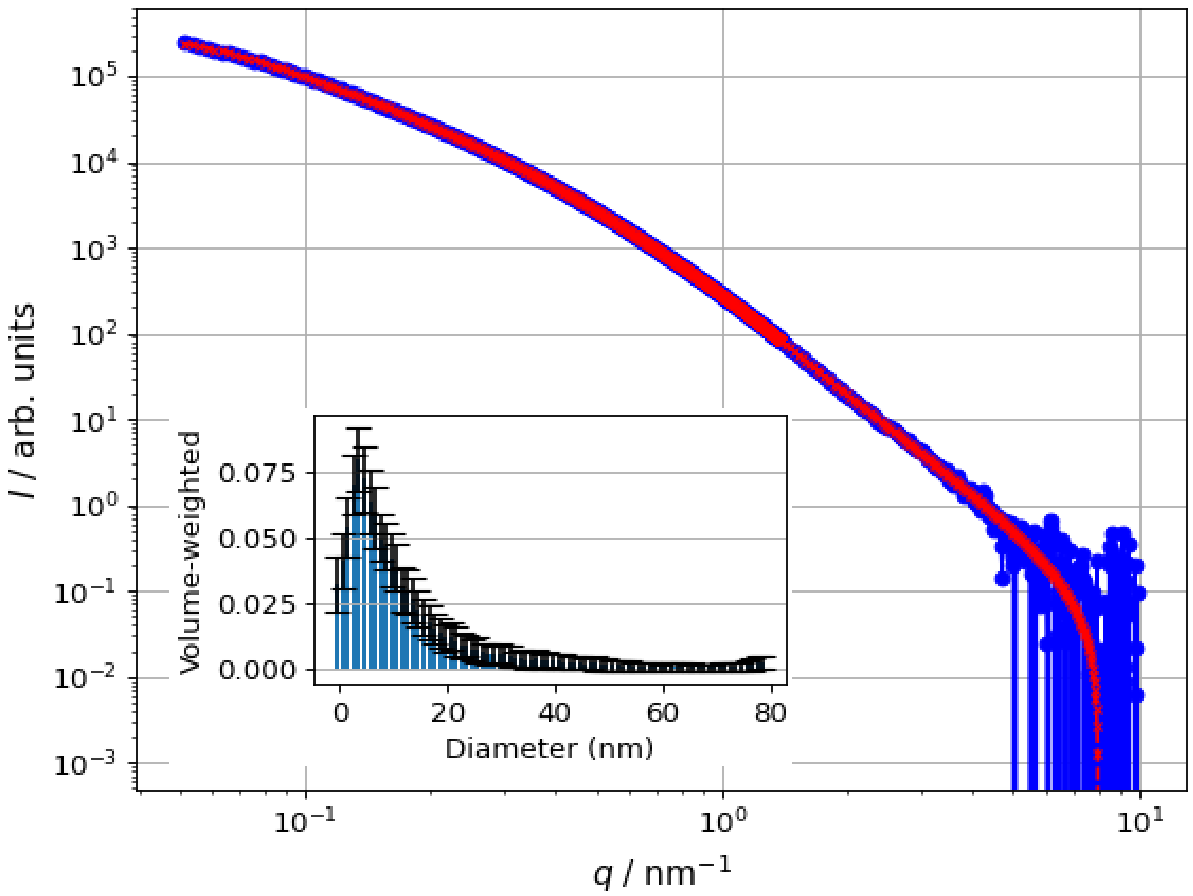

Figure SI3e- PTB SAXS data fitted using pysaxs, using the full q-range, almost surprisingly, the difference as compared to the analysis from the main text is marginal.

The median diameter is 8.1 nm and the mean diameter is 12.3 nm, and the maximum of the distribution is at 4.1 nm, all three identical to the results from the main article. The variance is 13.2 nm comparing to 13.0 nm from the main article. When fitting a Gaussian to the distribution, we get a mean value μ = 5.3 nm (5.4 nm in the main article) and the standard deviation 2σ = 15.0 nm (14.6 nm in the main article).

D) DLS data on lyophilized and redispersed FeraSpin ™ R colloids

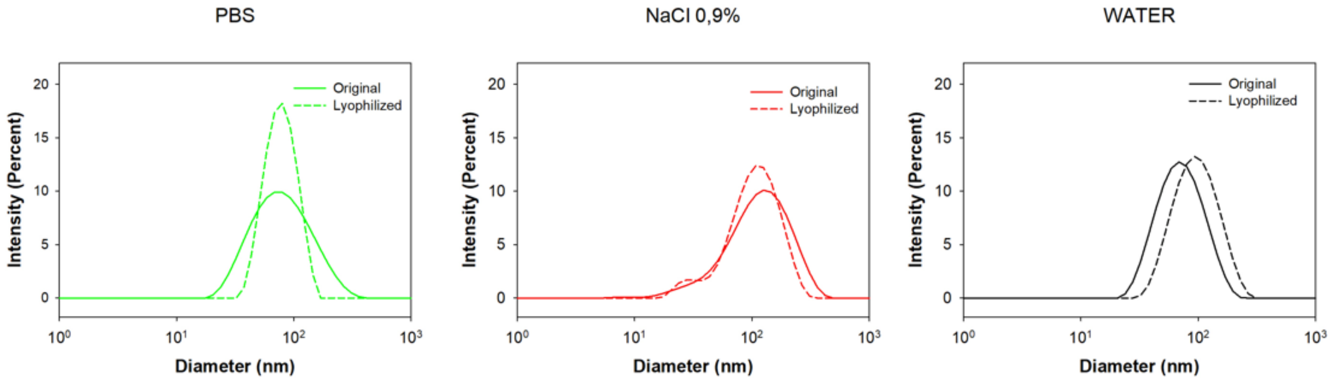

Figure SI4- DLS size distributions of lyophilized FeraSpin ™ R samples redispersed in different matrixes, compared to the original colloid diluted in the same matrixes.

Table SI2- DLS data on lyophilized and redispersed FeraSpin ™ R colloids

	H_2O	NaCl 0,9 %	PBS 0.01 M
**Size MADLS (nm)**	92 ± 5	99 ± 7	93 ± 5
**Size DLS 173° (nm)**	82 ± 3	89 ± 8	81 ± 4
**PDI**	0.200 ± 0.007	0.24 ± 0,02	0.215 ± 0.003
**ζ Potential (mV)**	-49 ± 2	-11 ± 2	-11.1 ± 0.9

DLS data on lyophilized and redispersed FeraSpin ™ R colloids together with the standard deviation of three independent measurements

E) Additional TGA data

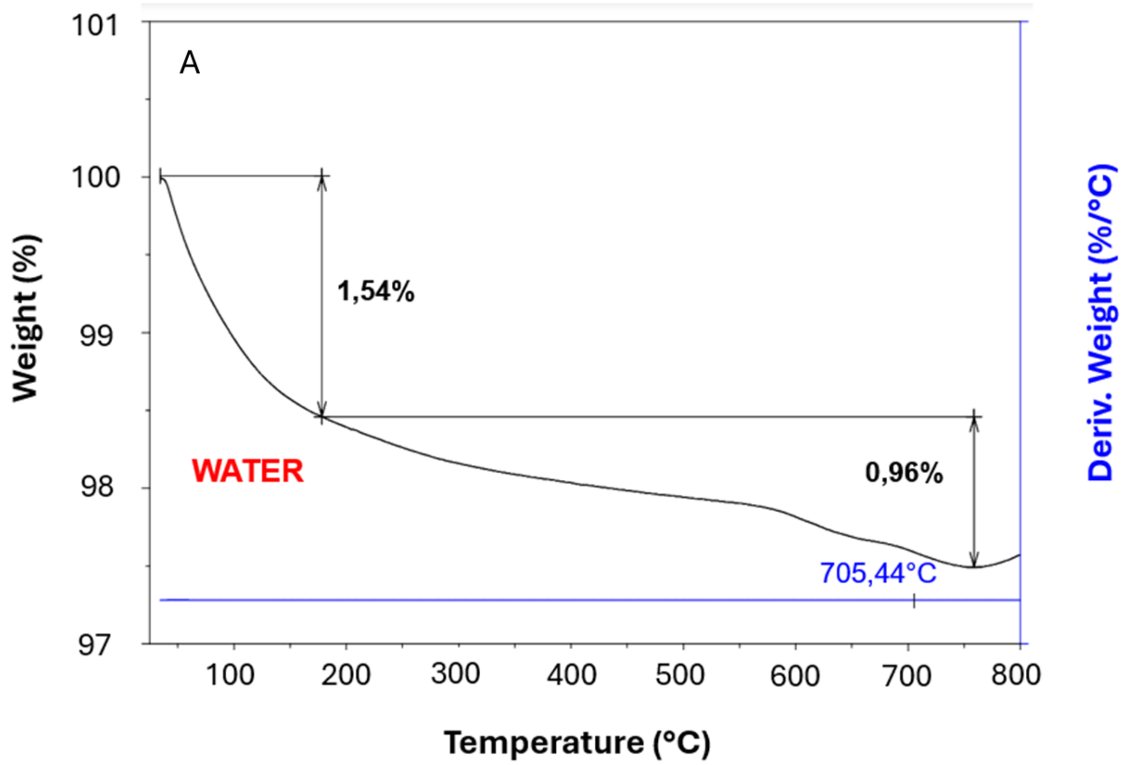

Figure SI5a- Thermogravimetric analysis of the sample Fe_2O_3 shown in a range from 97 % to -101 %

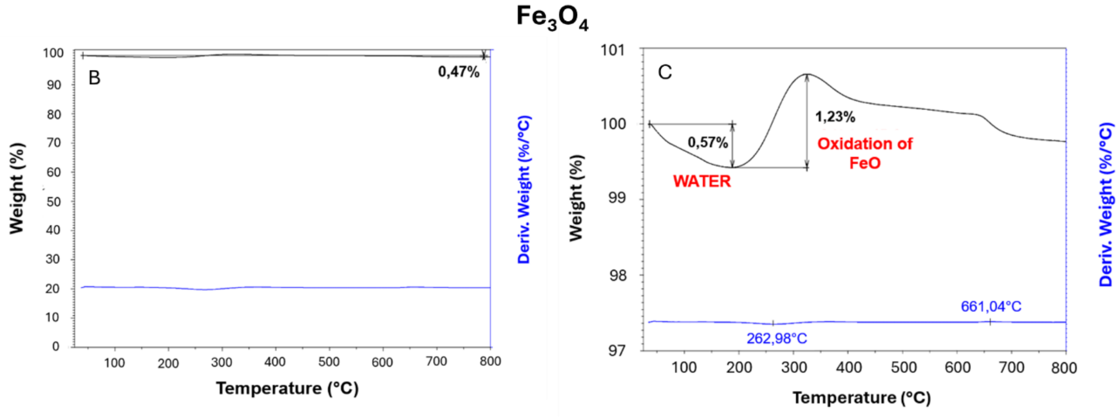

Figure SI5b- Thermogravimetric analysis of the sample Fe_3O_4 (B) full graphic and (C) shown in a range from 97 % to -101 %

F) ICP analysis of Feraspin ™ R aliquots

Table SI3- ICP-MS results obtained for the determination of $[Fe]_{tot}$. The sample was divided in 5 aliquots of 50 µL . Each aliquot was mineralized and analyzed by ICP-MS.

	**Fe mg/kg**
Mineralisation E1	975
Mineralisation E2	973
Mineralisation E3	978
Mineralisation E4	980
Mineralisation E5	990
mean	979
Standard deviation	6.7
RSD	0.68 %

G) ATR-FTIR spectra of iron oxides and FeraSpin ™ R

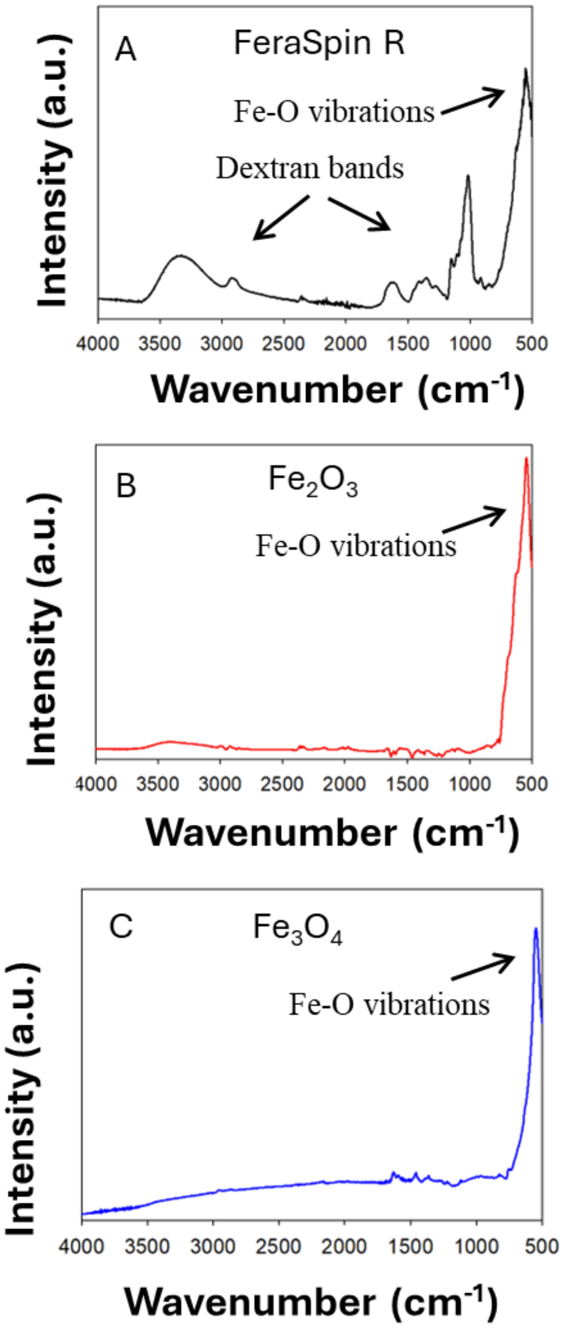

Figure SI6- FT-IR/ATR spectra of (A) Feraspin ™ R, (B) Fe_2O_3, (C) Fe_3O_4

H) Raman spectra of iron oxides and Feraspin ™ R

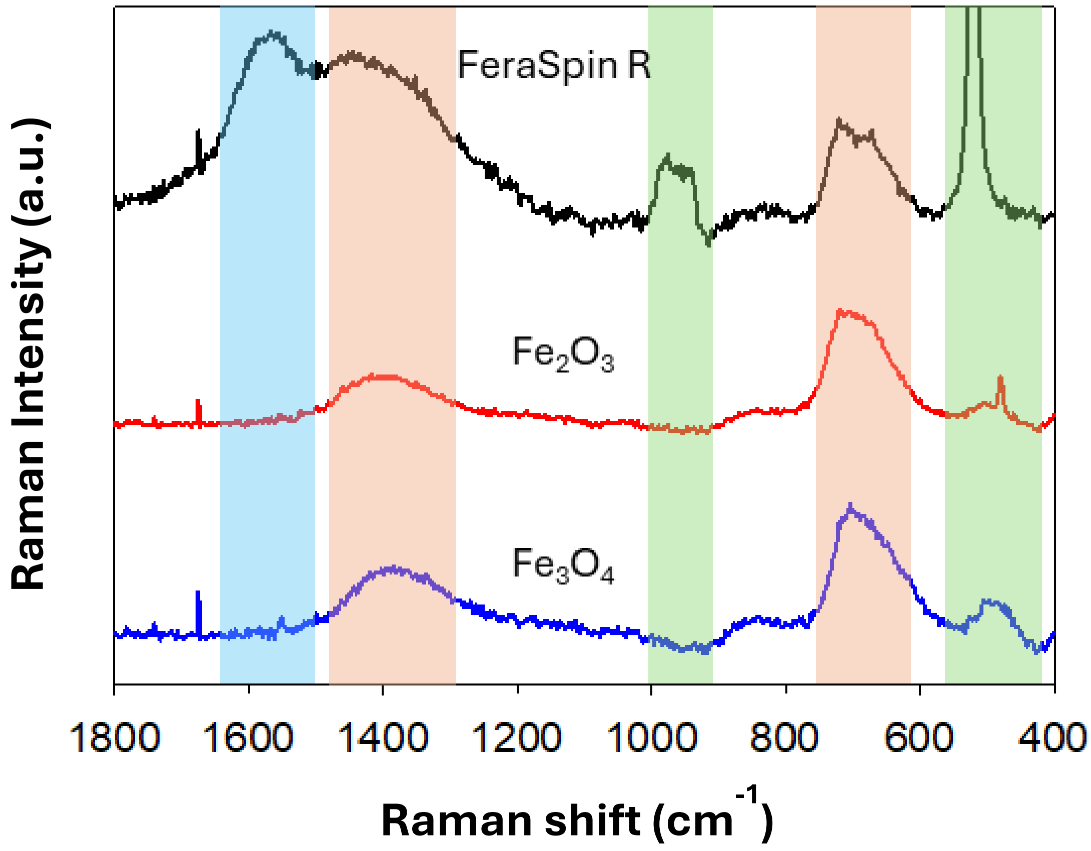

Figure SI7- Raman spectra of Feraspin ™ R (black), Fe_2O_3 (red) and Fe_3O_4(blue), green spot corresponds to silicon characteristic peaks, orange to iron oxide peaks and light blue to organic.

I) Key statistical parameters from chemical composition histograms obtained by micro-spectroscopy methods

Raman compositional histogram showed a single mode-distribution. Statistical measures were calculated from the intensity ratio histogram of the FeOx and organic coating Raman peaks.

Table SI4- Key statistical parameters from intensity ratio histogram for FeO_x and organic coating Raman peaks

Mean	0.43
Mode	0.48
Median	0.24
Variance	0.022
Standard Deviation	0.15
Skeweness	0.42
Kurtosis	6.32

SEM-EDX compositional histogram showed a bimodal distribution. Statistical measures were calculated for the two populations identified within histograms of the Fe/C ratio. A cut-off value at ratio 0.6 was selected, corresponding to the intercept point of the modal distributions. This threshold separates the first peak, accounting for 63 % of the population, from the second peak, comprising the remaining 37 %

Table SI5- Key statistical parameters from EDX atomic ration histogram for iron and carbon for the main modal distribution (relative abundance 63 %)

Mean	0.42
Mode	0.43
Median	0.29
Variance	0.007
Standard Deviation	0.08

Table SI6- Key statistical parameters from EDX atomic ration histogram for iron and carbon for the secondary modal distribution (relative abundance 37%)

Mean	0.78
Mode	0.78
Median	0.66
Variance	0.008
Standard Deviation	0.09

J)- XPS data of Feraspin ™ R

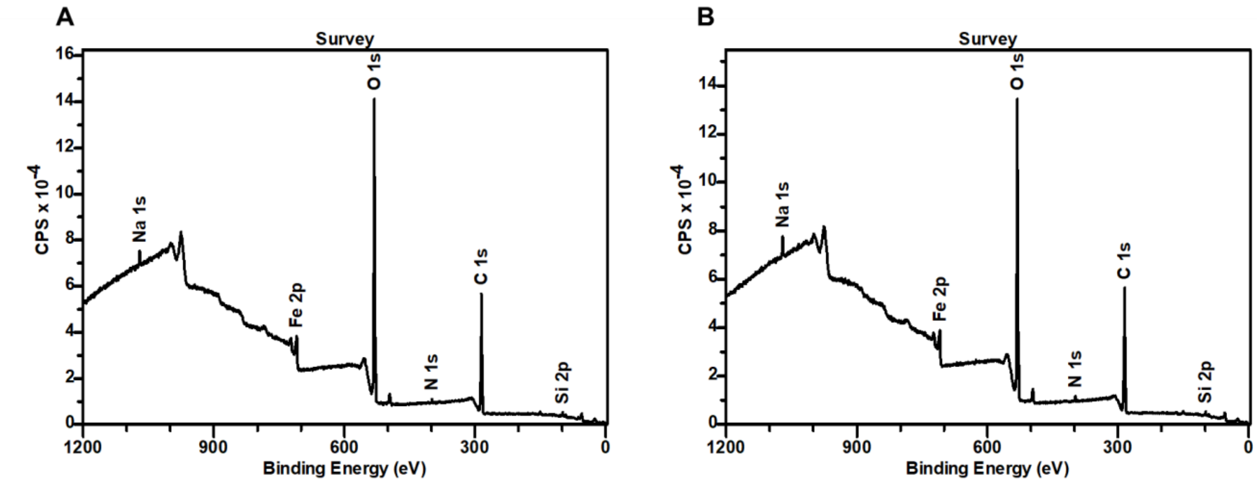

Figure SI8a- Representative XPS survey spectra of (A) vacuum-desiccated FeraSpin R, (B) air dried FeraSpin R.

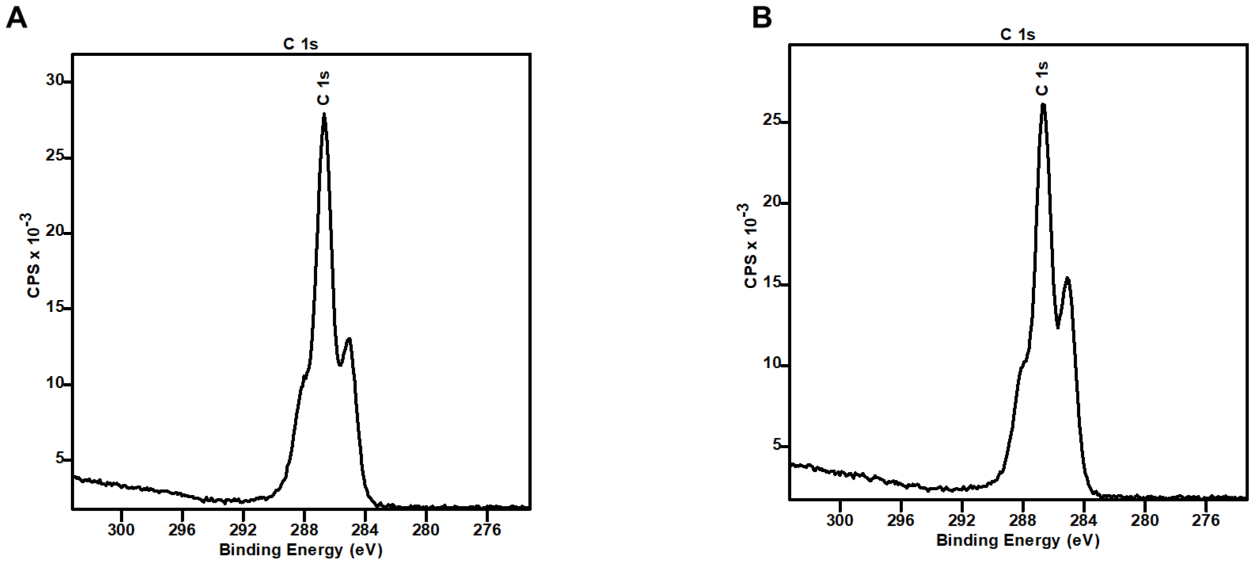

Figure SI8b - Representative XPS carbon narrow scans of (A) vacuum-desiccated FeraSpin R (sample 2E), (B) air dried FeraSpin R (sample 2A)

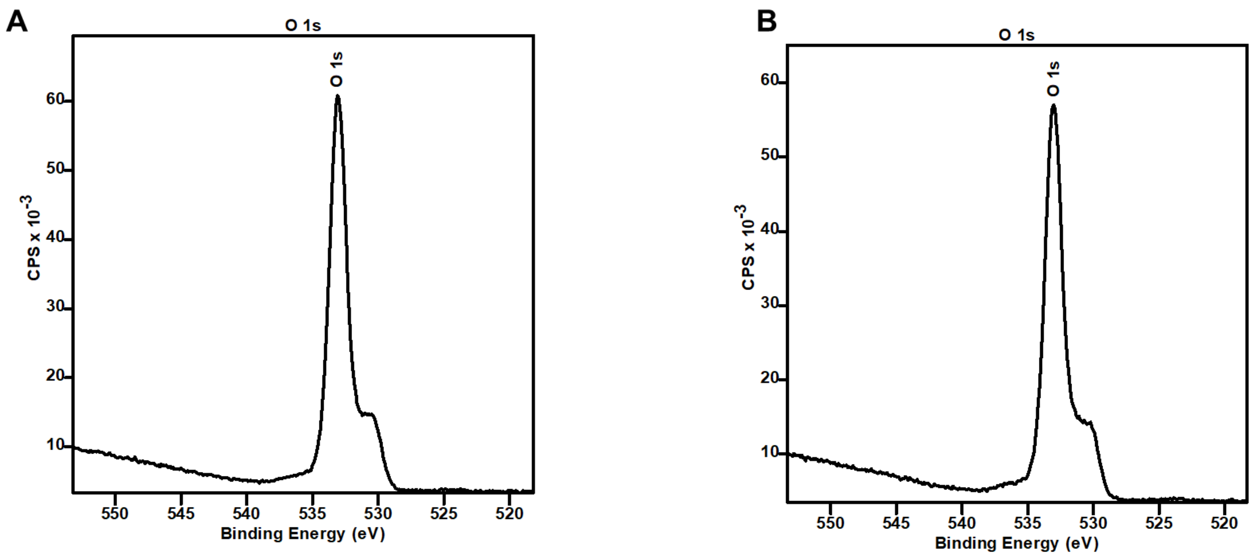

Figure SI8c- Representative XPS oxygen narrow scans of (A) vacuum-desiccated FeraSpin R (sample 2E), (B) air dried FeraSpin R (sample 2A).

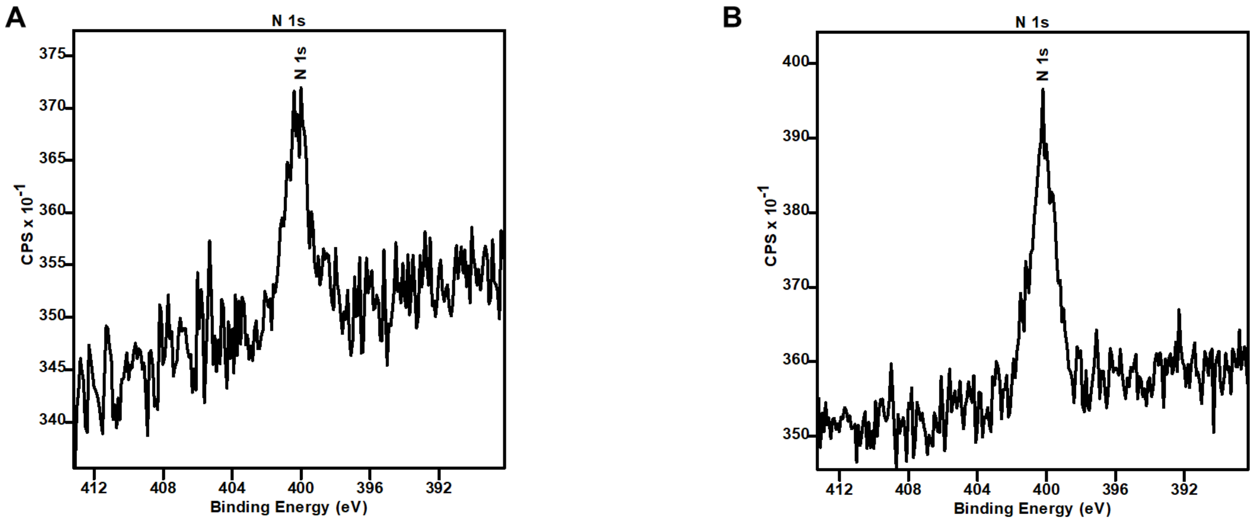

Figure SI8d- Representative XPS nitrogen narrow scans of (A) vacuum-desiccated FeraSpin R (sample 2E), (B) air dried FeraSpin R (sample 2A)

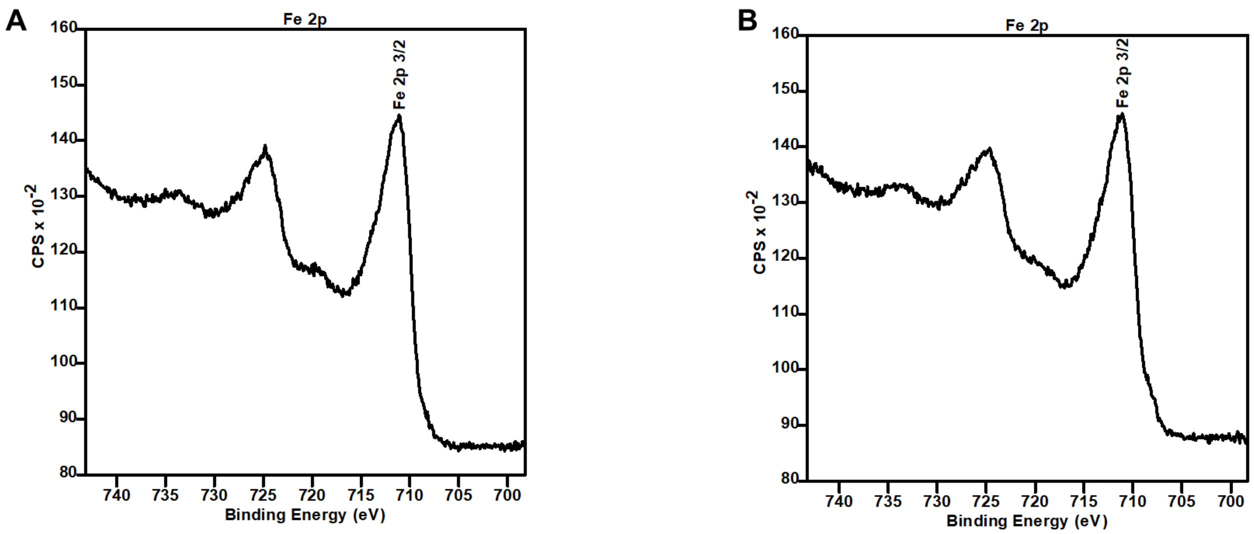

Figure SI8e- Representative XPS iron narrow scans of (A) vacuum-desiccated FeraSpin R, (B) air dried FeraSpin R

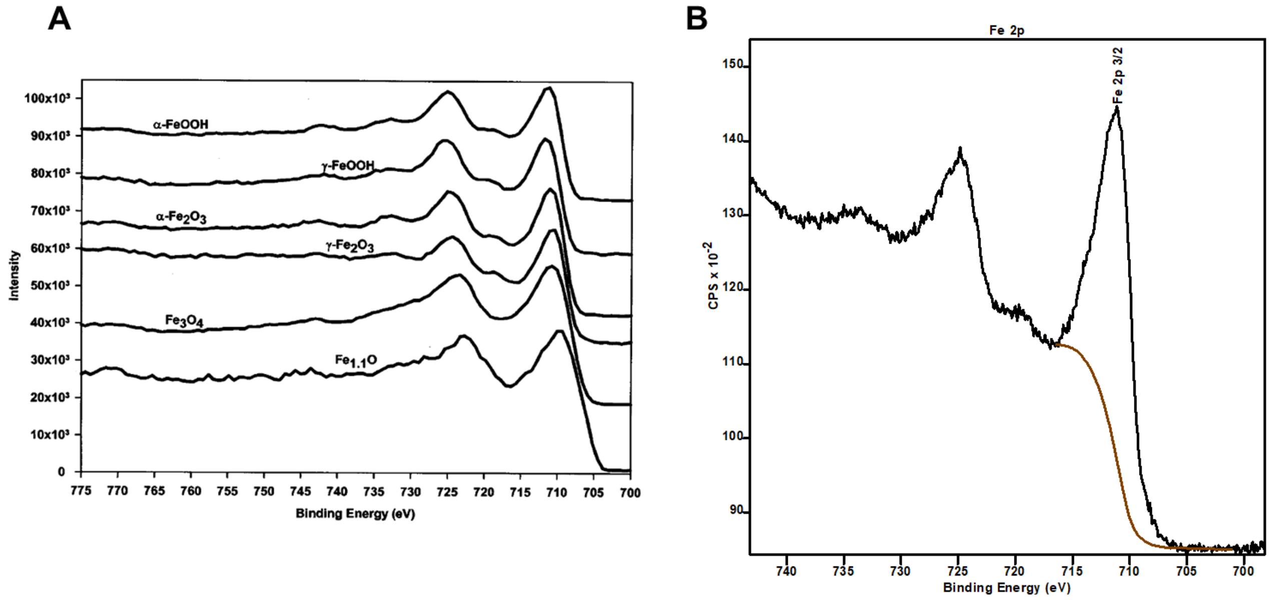

Figure SI8f- (A) Fe 2p peak spectra in literature, (B) Fe 2p peak with Shirley background on 2p 3/2 peak used as a quantity proportional to total Fe atomic %.

Table SI7- Atomic composition of different FeraSpin R aliquots by XPS analysis.

	**Atomic % per Sample**					
**Element**	**1E**	**2E**	**3E**	**1A**	**2A**	**3A**
**Na**	1.4	2.2	2.1	2.5	2.5	2.6
**Fe**	1.7	1.6	1.5	1.8	1.6	1.6
**O**	41.2	40.5	40.0	39.7	38.9	39.3
**N**	0.3	0.2	0.2	0.3	0.4	0.3
**C**	53.9	53.8	54.2	54.0	55.1	54.2
**Si**	1.6	1.8	2.1	1.7	1.6	2.0
**Fe/C**	0.032	0.030	0.028	0.033	0.029	0.030
**Fe/C mean**	0.030			0.031		
**Fe/C STD**	0.002			0.002		
**Fe/C RSD**	6.5 %			7.7 %		

#E refers to samples dried under vacuum within a desiccator.

#A refers to samples died in air.

Table SI8- Experimental details for XPS analysis and instrumental settings.

**Instrument and Parameters**	
**Instrument**	AXIS Supra^{+}
**Manufacturer**	Kratos Analytical Ltd
**X-ray source**	Monochromated Al Kα (1486.6 eV)
**X-ray anode settings**	15 kV; 5 mA (75 W)
**Analysis spot**	300 µm × 700 µm
**Survey spectra acquisition**	Pass energy: 80 eV<br>Step size: 0.5 eV<br>Sweep / dwell time: 732 s / 300 ms<br>Sweeps: 1
**Narrow spectra acquisition**	Pass energy: 40 eV<br>Step size: 0.1 eV<br>Target quality: 200 (signal-to-noise)<br>Max sweeps: 10
**Charge neutraliser**	Filament current: 0.45 A<br>Filament bias: 1.0 V<br>Charge balance: 4 V
**Lens Mode / Aperture Setting**	Hybrid / Slot
**Acquisition Software**	ESCApe™
**Analysis Software**	CasaXPS (v. 2.3.25), MS Excel

K)- Uncertainty value on the main size by SEM estimation

The given uncertainty value on the main size is calculated with the propagation of the associated uncertainties of the influencing parameters on the measurand for k=2. In this case, the measurand D_{eq} corresponds to the equivalent diameter in projected surface. The influencing parameters on the measurand and their associated uncertainty values u_i^2 are summarized in Table SI6. Thus, the uncertainty value on the main size for k=2 ($U\ \left(D_{eq}\right)$) is estimated with Eq0.

Table SI9: Influencing parameters on the measurand and their associated uncertainty values u_i^2

Influencing parameters	Symbols	Values (u_i^2)
Beam size	$u_{tf}\ (D_{eq})$	2.89
Sampling	$u_{ech}\ (D_{eq})$	1.27
Instrument drift	$u_d\ (D_{eq})$	0.00953
Calibration	$u_c\ (D_{eq})$	1.33e-6

$$U\ \left(D_{eq}\right) = 2 * u\ \left(D_{eq}\right) = 2 * \sqrt{u_{tf}^2 +\ u_{ech}^2 + u_d^2 + u_c^2} \qquad \text{EqS0}$$
